# Genesis mechanisms of medium- and low- temperature convective geothermal fields in Wenquan County, Xinjiang

**DOI:** 10.1371/journal.pone.0354385

**Published:** 2026-08-06

**Authors:** Chenhao Sun, Zhilong Qi, Zizhao Zhang, Kai Chen, Ying Xi, Yalu Wang, Mengmeng Zhao

**Affiliations:** 1 College of Geology and Mining Engineering, Xinjiang University, Urumqi, China; 2 Key Laboratory of Green and Efficient Mining and Ecological Restoration of Mines in Alpine and Arid Areas of Xinjiang, Urumqi, China; 3 Hydrological and Environmental Geological Survey Center of Geological Bureau of Xinjiang Uygur Autonomous Region, Urumqi, China; National Autonomous University of Mexico Institute of Geophysics: Universidad Nacional Autonoma de Mexico Instituto de Geofisica, MEXICO

## Abstract

The area around Wenquan County, Xinjiang, is home to numerous hot springs and abundant geothermal resources, offering significant potential for development and utilization. Currently, systematic research on the formation mechanisms of geothermal fields in this region remains relatively limited, which to some extent constrains the comprehensive development and utilization of Wenquan County’s geothermal resources. This study selected the area around Wenquan County as the study area and employed methods such as remote sensing interpretation, ground temperature measurement, geophysical exploration and drilling, geothermal gradient calculations, rock radioelement measurements, hydrochemical analysis, and isotope analysis to elucidate the genesis mechanisms of the geothermal field. The results indicate that: (1) The geothermal anomaly areas in the Wenquan County geothermal field are primarily controlled by fault structures such as F2, F4, F3-1, and F17. The average geothermal gradient reaches 11.53°C/100 m, and the heat flow value of 320 mW/m^2^ is significantly higher than the average heat flow value at the northwestern margin of the Junggar Basin in Xinjiang, indicating a favorable terrestrial heat flow background. (2) Within the study area, well-developed structural fissures formed at the intersections of F2, F17, and F3-1, coupled with fractured rock masses, provide favorable conditions for groundwater storage and hydrothermal exchange. Based on the silica geothermometer, the average reservoir temperature is estimated at 132.4°C. Analysis indicates that the geothermal reservoir is located within structurally fractured zones, extending in a strip-like pattern on the surface, and belongs to the medium-low temperature belt-type geothermal reservoir. (3) The geothermal water in the study area has a SO_4_-Na hydrochemical type, originating from the infiltration of atmospheric precipitation in the southern Biezhentao Mountain area. It has undergone intense water-rock interactions, including hydrothermal alteration, sulfide oxidation, and decarbonation, transporting deep-seated heat to the surface through fault fracture zones. This study has essentially clarified the genesis model of the Wenquan County geothermal field, providing a crucial basis for the development and utilization of geothermal resources.

## 1. Introduction

As low-carbon, clean, and renewable geothermal resources, they can not only effectively reduce reliance on fossil fuels but also significantly lower greenhouse gas emissions, making them of vital strategic importance to the global energy transition and sustainable development. At the same time, accelerating the development and utilization of geothermal energy is also one of the indispensable pathways to achieving the “dual carbon” goals. Therefore, research into the genetic mechanism of geothermal resources serves as the theoretical foundation for geothermal resource exploration and development; it determines the accuracy of delineating geothermal anomaly areas and directly impacts exploration risks and costs.

Research on the genetic mechanism of geothermal resources aims to elucidate the sources, accumulation, and transport processes of thermal energy within the Earth, thereby providing a scientific basis for resource assessment and development. Currently, scholars both domestically and internationally have conducted extensive research on the formation mechanisms of geothermal systems. In terms of geochemical tracing, previous studies have analyzed the geochemical characteristics of geothermal fluids to reveal their sources, water-rock interactions, tectonic control features, and the mechanisms by which fluids ascend along fractures [1,2]. Regarding geological structures and heat control mechanisms, domestic scholars have primarily focused their research on four key aspects: “heat source, reservoir, conduit, and overburden” [3]. By comprehensively employing methods such as geological structural analysis, temperature field and hydrochemical characteristic analysis, interpretation of fracture systems and groundwater circulation pathways, regional tectonic evolution analysis, deep geophysical exploration, thermodynamic evaluation of geothermal reservoirs and overburden, geochemical temperature scales and analysis of radiogenic heat production rates, hydrogeological mapping, and remote sensing interpretation. These studies have revealed the genesis mechanisms of different types of geothermal fields, established a “source-pathway-reservoir-cap” integrated model, and elucidated heat generation mechanisms such as “western precipitation recharge—deep thermal conduction—structural fissure conduction—sedimentary layer heat retention” [4–9].

Xinjiang possesses a diverse range of geothermal resources, including not only shallow geothermal resources, medium-to-deep hydrothermal geothermal resources, and hot dry rock (HDR) geothermal resources, but also newly identified oil and gas-associated geothermal resources and coal spontaneous combustion-type geothermal resources [10]. Due to the relatively dispersed spatial distribution of geothermal resources in Xinjiang, coupled with factors such as poor transportation infrastructure and a scattered population, the utilization of these resources remains lower compared to other inland regions of China, and related research is still in its early stages [11–13]. However, in recent years, with advancements in science and technology, significant progress has been made in geothermal research in Xinjiang. In terms of remote sensing interpretation and geothermal target area delineation, previous researchers have successfully identified viable geothermal target areas through methods such as hydrogeological remote sensing interpretation and land surface temperature retrieval, and have determined the extent of geothermal fields, reservoir temperatures, resource reserves, and the chemical characteristics of geothermal fluids [14,15]. In terms of geophysical exploration and fault structure identification, previous researchers comprehensively employed various geophysical methods, such as AMT and EH-4, in conjunction with geological surveys and drilling operations. This enabled the accurate determination of fault locations and rock-rock contacts, elucidated the mechanisms of geothermal generation, and established the genesis and extent of geothermal fields [16,17]. In the field of geochemistry and hydrogeology, previous researchers employed methods such as the Shchukarev classification and Piper trilinear diagram. Through comparative analysis of groundwater composition and chemical characteristics, they identified the hydrochemical types of geothermal resources, their recharge sources, and the types of geothermal reservoirs [18].

In summary, while advanced technical methods and systematic research approaches have been established for studying the genesis mechanisms of geothermal resources both domestically and internationally, research on multi-source coupling in the Xinjiang region remains relatively underdeveloped. Except for the Shengshan Geothermal Field, exploration in other areas of Wenquan County, Xinjiang, is generally at a low level, which imposes certain limitations on the development and utilization of geothermal resources within the study area. Therefore, this study focuses on the Wenquan County geothermal field. Based on a systematic analysis of the geothermal geological conditions in the study area, it examines the formation mechanisms of geothermal resources using methods such as geophysical exploration and drilling, remote sensing interpretation, geochemical exploration, hydrogeochemistry, and isotope analysis, thereby providing a scientific basis for the development and utilization of geothermal resources in Wenquan County.

## 2. Overview of the study area

**Fig 1:**
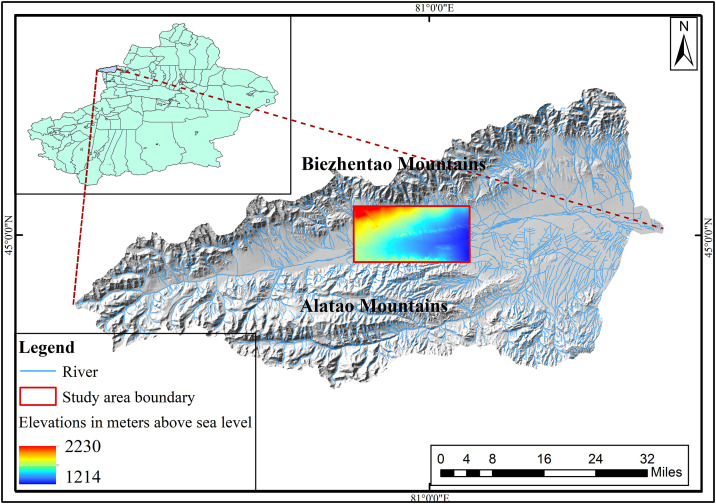
Location map of the study area.

The study area is located in an intermontane valley between the Biezhentao Mountains and the Alatao Mountain. The general topography slopes from west to east and from north to south, with the terrain on both the northern and southern sides sloping toward the center. Elevations range from 1214 ~ 2230 m. The location of the study area is shown in Fig 1. The geomorphic units within the study area include tectonic erosion and weathering landforms and sedimentary landforms. The tectonic erosion and weathering landforms comprise eroded and weathered mid-mountain areas, eroded and weathered low mountains and hills, and ridge-and-plateau-like eroded terraces. The sedimentary landforms include alluvial-fluvial sloping plains and alluvial plains.

### 2.1. Regional geothermal geological conditions of the study area

Geologically, the geothermal fields in the study area are classified into the Junggar-Northern Tianshan Fold System (II) and the Tianshan Fold System (III) as primary tectonic units, and the Northern Tianshan Yudi Trough Fold Belt (II_3_) and the Borokonu Trough Fold Belt (III_1_) as secondary tectonic units. The distribution of igneous rocks in the region includes granites and vein rocks, primarily consisting of Late Mesoproterozoic granites, Mid-Variscan granites, and vein rocks. Gneissic granites are widely developed, with zircon Pb^206^/U^238^ apparent ages ranging from 926 ~ 1100 Ma. The region and its surroundings feature well-developed tectonic layers from multiple cycles, including the Wutai, Tarim, Hualixi, and Himalayan cycles, along with the distribution of Meso-Paleozoic and Middle-to-Late Hualixi neutral-to-acidic intrusive rocks. Fault structures are highly developed within the region; their formation is closely related to the corresponding tectonic movements of the Wutai, Tarim, Hualixi, and Himalayan cycles, and they are all products of the corresponding tectonic movements of those periods. These fractures cut through the granites and connect to deep heat sources, causing rock fragmentation and providing the conditions and prerequisites for the migration and accumulation of geothermal fluids and the formation of geothermal systems.

### 2.2. Geothermal geological conditions of the geothermal fields in the study area

**Fig 2:**
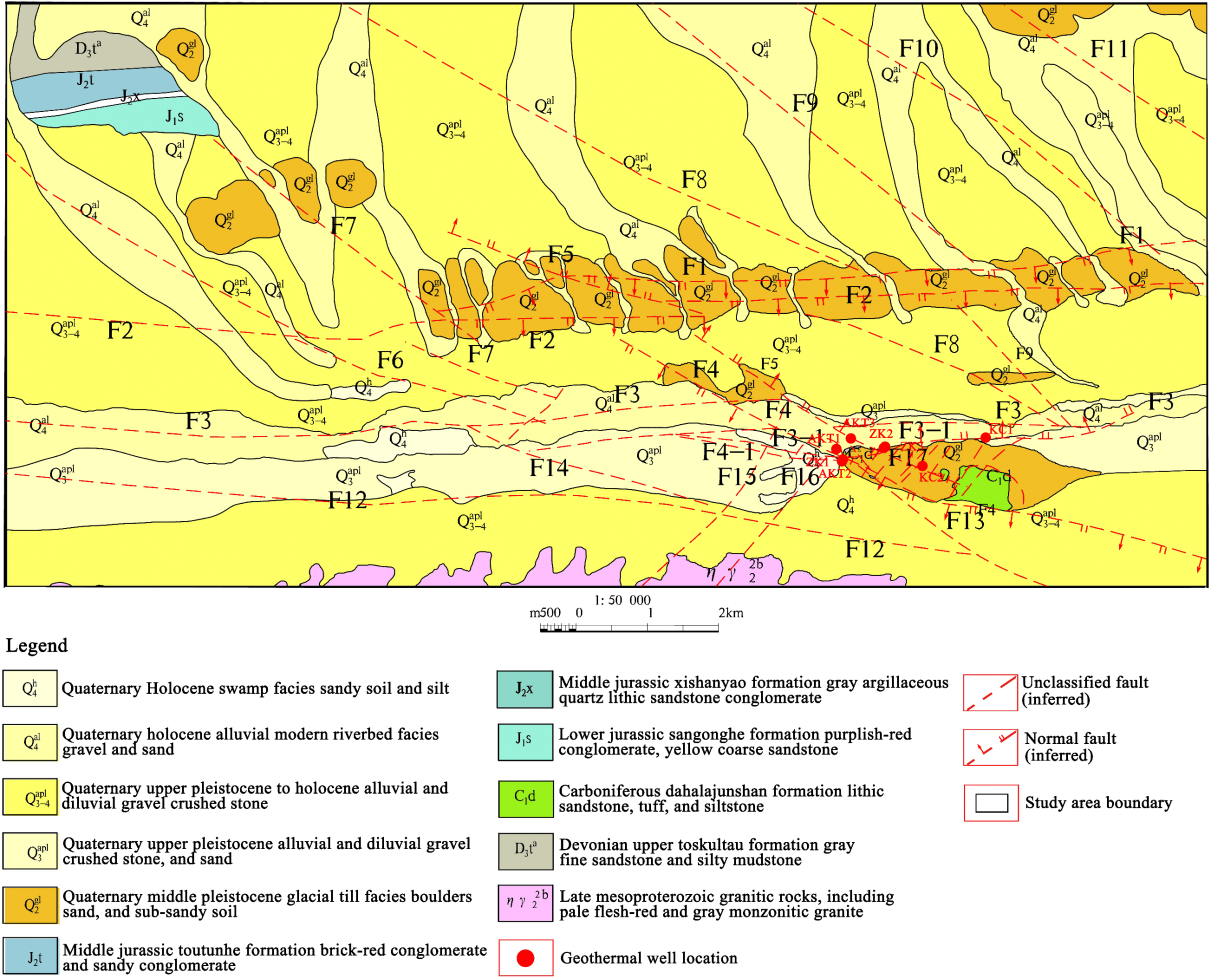
Geological map of the study area (Reproduced with permission from an internal technical report of the Xinjiang Geological and Mineral Bureau First Hydrogeological Engineering Brigade [19]).

Geologically, the geothermal field in the study area is situated at the intersection of the EW-trending faults F1, F2, F3, and F3-1, and the NE-SW-trending faults F15, F16, and F17. The EW-trending faults extend along the Bortala River, the Modun Uplift, and the Shengshan Uplift. The main and secondary faults F2 and F3 are well-developed, and the degree of fracturing and water saturation of the strata varies greatly; the NE-SW-trending faults are distributed within the Shengshan Uplift and to its west, intersecting and closely related to faults F3, F3-1, and F4, and are major water-conducting faults. Hot springs are found at the intersection of the F17 fracture group and the F3-1 fracture [19]. Groundwater in the study area is primarily bedrock fissure water, which can be classified into two types: interbedding fissure water and vein-type fissure water. The interbedding fissure aquifer consists mainly of volcaniclastic sedimentary rocks, with a thickness exceeding 700 m, a permeability coefficient of 0.01 ~ 0.487 m/d, and a single-well yield of 800 ~ 1538.78 m^3^/d, indicating moderate water-bearing capacity; Vein-type fissure water is found in fracture zones and fractured veins and constitutes the primary aquifer in the study area. Within an exploration depth of 1000 m, the aquifer consists of Paleozoic sedimentary, metamorphic, and igneous rocks with well-developed structural fissures. The stable natural flow rate ranges from 4909.03 ~ 7700.09 m^3^/d, indicating high water yield. Groundwater recharge primarily originates from the infiltration of atmospheric precipitation from the Biezhentao Mountains in the south and the Alatao Mountain in the north. It converges and circulates within the structural fractures before ultimately flowing toward the eastern plains. The maximum temperature of spring water in the area is 70.1°C, while the water temperature from geothermal wells ranges from 52.3 ~ 62.4°C, with a maximum stable flow rate of 7700.09 m^3^/d. This indicates that the study area possesses favorable geothermal geological conditions and that its geothermal resources have significant potential for development and utilization. The geological map of the study area is shown in Fig 2.

## 3. Methods and data

**Fig 3:**
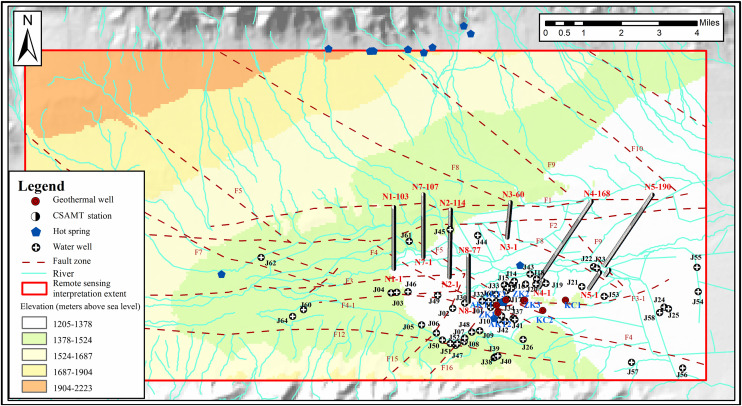
Work deployment map for the study area.

This paper analyzes the geothermal genesis mechanisms in the study area primarily through methods such as remote sensing interpretation, ground temperature measurement, geophysical exploration and drilling, geothermometer calculations, rock radioelement measurements, and hydrochemical and isotope analyses. The specific work layout is shown in Fig 3.

### 3.1. Ground temperature measurement

Ground temperature measurements provide direct information on the ground temperature field and are the most direct approach for studying geothermal geological conditions. This paper primarily employs remote sensing technology and field measurements to conduct ground temperature surveys in the study area. Remote sensing technology uses surface temperature inversion methods to obtain comprehensive temperature data for the study area, thereby providing a macro-level and accurate representation of the extent of thermal anomalies. Field ground temperature measurements involve collecting temperature data from geothermal wells in the study area to generate isotherm maps, enabling precise analysis and verification of the centers of thermal anomalies and their surrounding diffusion patterns. The specific procedures are described as follows.

(1) Remote sensing technology

**Fig 4:**
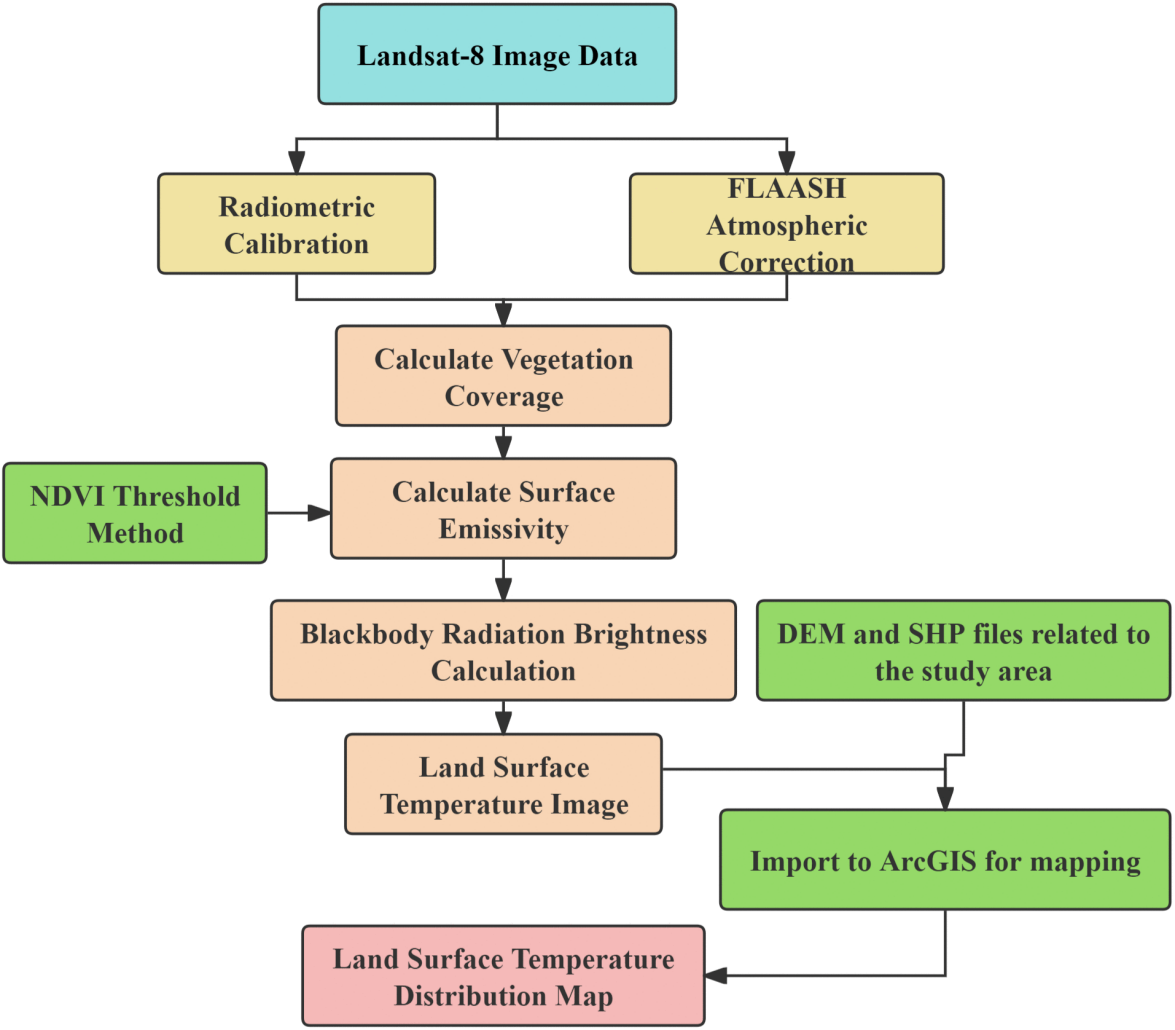
Remote sensing interpretation flow chart.


Remote sensing technology characterizes ground temperature anomalies by detecting thermal radiation from the Earth’s surface. It offers not only the advantage of wide coverage but also high efficiency and low cost, and is widely used in identifying geothermal anomaly zones and delineating exploration targets [20]. In this study, Landsat-8 thermal infrared remote sensing imagery was selected, and ENVI and ArcMAP software were used to perform surface temperature inversion in the study area, thereby providing comparative support for geothermal research. The specific research formula is as follows, and the flowchart is shown in Fig 4 [21]:

$$\begin{array}{c} L_{\lambda }=[\varepsilon \cdot B(T_{s})+(\text{1}-\varepsilon )L_{↓}]\cdot \tau +L^{↑} \end{array}$$ (1)

In the equation:*ε*is the surface emissivity; *T*_*S*_ is the true surface temperature;*τ*is the atmospheric transmittance in the thermal infrared band; *L*^↑^,*L*^↓^ are the atmospheric upward/downward radiance.

The expression for the radiant intensity B(TS) of a blackbody at temperature T in the thermal infrared band is as follows:


$$\begin{array}{c} B(T_{S})=[L_{\lambda }-L^{↑}-\tau (\text{1}-\varepsilon )L_{↓}]/(\tau \cdot \varepsilon ) \end{array}$$ (2)

The actual surface temperature Ts is obtained using the inverse of Planck’s law, as expressed below:


$$\begin{array}{c} T_{S}=K_{\text{2}}/ln(\frac{K_{\text{1}}}{B(T_{s})}+\text{1}) \end{array}$$ (3)

(2) Field ground temperature measurement

Field ground temperature measurements are the most fundamental and critical component of geothermal resource exploration and development, as they provide direct access to accurate temperature data from various media, including geothermal boreholes, rocks, and soil. This study conducted ground temperature measurements at 63 water wells within the study area, with the survey layout shown in Fig 4. The SYKJ-9 deep-well electronic temperature logger was used for temperature measurement, with a range of −50 ~ 150°C. The instrument has passed testing by relevant quality inspection departments, ensuring the accuracy of the data collected during this measurement campaign.

The geothermal gradient is the rate of temperature change with depth below the Earth’s isotherm, generally expressed as the temperature increase per 100 m. The geothermal gradient is primarily influenced by the regional background geothermal flux and variations in the thermal conductivity of near-surface rock masses. It is also affected by factors such as lithological changes, groundwater circulation, and magmatic activity; therefore, the presence of geothermal flux can be identified based on anomalies in the gradient [22].

### 3.2. Thermal conductivity of rocks and terrestrial heat flow

Rock thermal conductivity is a thermal property parameter that characterizes a rock’s ability to conduct heat; it is an indispensable parameter for studying regional terrestrial heat flow and the mechanisms underlying geothermal genesis [23]. This study involved testing the thermal conductivity of rock cores extracted from geothermal boreholes, with a total of 120 sets of cores representing different lithologies. The testing was conducted by the Hydrogeological and Environmental Survey Center of the Xinjiang Uygur Autonomous Region Geological Bureau using a KD2 Pro soil thermal properties analyzer. The transient hot-wire method was employed to determine thermal conductivity by measuring the thermal response of the probe within the rock samples.

Geothermal flux is the amount of heat transferred from the Earth’s interior to the air at the Earth’s surface per unit area and per unit time. It reflects the thermal state of the deep lithosphere at the Earth’s surface, and research on geothermal flux values is a fundamental and critical component of geothermal studies [24]. Under ideal conditions of pure heat conduction without internal heat sources, geothermal flux can be calculated using Fourier’s law [25,26]:


$$\begin{array}{c} q=K\frac{dt}{dh} \end{array}$$ (4)

In the equation: *K* is the thermal conductivity of the rock, W/(m·K); *dt/dh* is the vertical temperature gradient, °C/km.

### 3.3. Rock radiogenic heat production rate

Radioactive elements are commonly found in various naturally occurring rocks; through their decay processes, these elements release large amounts of heat, making them one of the primary sources of heat within the lithosphere [27]. The radiogenic heat production rate of a rock is a key parameter for assessing its radioactive heat-producing capacity. It can be calculated by measuring the concentrations of uranium, thorium, and potassium in the rock and applying the formula proposed by Rybach [28], which is based on corrected natural radioactive nuclear parameters.


$$\begin{array}{c} A={\text{10}}^{-\text{5}}\rho (\text{9}.\text{52}C_{U}+\text{2}.\text{56}C_{T\text{h}}+\text{3}.\text{48}C_{K}) \end{array}$$ (5)

In the equation: *A* is the rock’s radioactive heat generation rate, μw/m^3^; *ρ* is the rock’s density, kg/m^3^; *C*_*U*_, *C*_*Th*_, *C*_*K*_ represent the rock’s U(ug/g), Th(ug/g), K(%) content, respectively.

Granite bodies are widely distributed within the study area, and granite-type rocks typically contain high concentrations of radioactive elements such as U, Th, and K [29]. In this study, 43 sets of rock core samples of different lithologies were collected from four geothermal wells—KC2, AKT1, AKT2, and AKT3—to conduct tests for uranium, thorium, and potassium concentrations. The testing was conducted by the Testing and Research Institute of the 216th Brigade of the Nuclear Industry. Using an ICP-MS instrument and the standard solution calibration method, quantitative analysis was performed on liquid samples that had undergone digestion and pretreatment to determine their U, Th, and K concentrations.

### 3.4. Geophysical exploration and drilling

The bedrock granite in the study area exhibits high resistivity and poor electrical conductivity, whereas fault zones display distinct low-resistivity anomalies. Therefore, electromagnetic sounding can be used to analyze the location and size of fault zones within the study area. Controlled-source audio-frequency magnetotellurics (CSAMT) is widely used in geothermal exploration due to its advantages of deep penetration, high resolution, and strong resistance to interference [30]. Consequently, the Controlled-Source Audio-Magnetotelluric (CSAMT) method was selected for this geophysical study. Field data were collected using the GDP-32^II^ multifunctional geophysical workstation CSAMT data acquisition system from the United States. A total of eight geophysical survey lines were established, with the layout shown in Fig 4.

Based on the distribution characteristics of faults and thermal anomalies in the study area, a total of 8 boreholes were drilled (as shown in Fig 4). Among these, ZK1, ZK2, ZK3, AKT1, AKT2, and AKT3 were used to determine the deep locations of faults such as F4 and F17 and to investigate their thermal and hydraulic control characteristics, while KC1 and KC2 were used for geothermal resource extraction. Rock samples were obtained through core recovery from the boreholes, and physical and thermal properties of the rocks were measured. Combined with borehole temperature measurements, these data provide a foundation for research on the heat sources and thermal reservoirs of the geothermal field.

### 3.5. Geothermometer calculation

Geothermometers are one of the most widely used, cost-effective, and indirect methods for estimating the temperature of deep geothermal reservoirs during the early exploration phases. The principle behind them is that the chemical concentrations or concentration ratios of hot water are related to the temperature of the underground geothermal reservoir, and that geothermal fluids and minerals reach equilibrium under specific temperature and pressure conditions [31]. Therefore, before applying geothermometers, it is first necessary to determine whether the water-rock reaction has reached a state of equilibrium. This study employs the Na-K-Mg triangular diagram proposed by Giggenbach to assess the equilibrium state of water samples from the study area [32]. Based on the positions of the water sample points on the Na-K-Mg triangular diagram, the geothermal system was divided into three zones: full equilibrium water, partial equilibrium water, and immature water. Through this equilibrium assessment, the applicability of different types of cationic geothermometers can be determined: full equilibrium water is suitable for all cationic geothermometers; partial equilibrium water is suitable for the K-Mg geothermometer; and immature water is not suitable for cationic geothermometers and can only use the SiO_2_ geothermometer.

Considering that the geothermal water in the study area is weakly alkaline, with temperatures not exceeding 70°C, and that there are no hot spring eruptions at the surface, this study employs the quartz geothermometer, which accounts for no steam loss [33]. The calculation formulas for the three thermometers the silica geothermometer, the Na-K geothermometer [34], and the K-Mg geothermometer [32] are as follows:


$$\begin{array}{c} t=\frac{\text{1309}}{\text{5}.\text{19}-lg({SiO}_{\text{2}})}-\text{273}.\text{15} \end{array}$$ (6)

$$\begin{array}{c} t=\frac{\text{1217}}{\text{1}.\text{48}+lg({Na}^{+}/K^{+})}-\text{273}.\text{15} \end{array}$$ (7)

$$\begin{array}{c} t=\frac{\text{1309}\text{4410}}{\text{14}.\text{0}-lg(K^{+}/{Mg}^{\text{2}+})}-\text{273}.\text{15} \end{array}$$ (8)

In the equation: *t* represents temperature, in °C; *K*^*+*^, *Na*^*+*^, *Mg*^*2+*^, and *SiO*_*2*_ represent the concentrations of potassium ions, sodium ions, magnesium ions, and silicon dioxide, respectively, in mg/L.

### 3.6. Water sampling and testing

Research on the hydrogeochemical characteristics of geothermal fluids is an effective means of understanding the formation mechanism, hosting environments, and circulation processes of geothermal resources. In this study, water samples were collected from a total of eight geothermal wells within the study area, and the hydrochemical characteristics of the geothermal water were analyzed graphically using Piper diagrams [35]. Concurrently, tests for stable isotopes (δ^18^O and δD) and radioactive isotopes (tritium) were conducted on water samples from 27 sampling points (including wells and springs) within the study area. Isotope analysis was employed to provide data support for determining the sources and recharge mechanisms of groundwater in the study area [36]. Isotope samples were collected using the submerged sealing method: sample bottles were fully immersed in the water, and after filling, the lids were sealed below the water surface without leaving any air gaps. The collected samples were transported to the laboratory within 12 hours. Quality control for sample collection and analysis was conducted in accordance with “Methods for the Analysis of Groundwater—Part 2: Collection and Preservation of Water Samples” (DZ/T 0064.2–2021) [37], and primarily included the following measures:(1) Field blanks: Ultrapure water was used as blank samples and treated at the sampling site under the same collection, preservation, and transportation conditions as the actual samples, then delivered to the laboratory together with the actual samples. At least one field blank was included per batch to verify whether contamination occurred during the entire sampling and transportation process.(2) Field duplicates: Duplicate samples were collected under identical sampling conditions; the sampler was shaken during collection to ensure sample homogeneity. Field duplicates accounted for approximately 10% of the total samples, with at least one pair of duplicates per batch. The relative deviation between duplicate measurements was controlled within ±5%. Both the hydrochemical analysis and hydrogen and oxygen isotope testing were conducted by the Hydrogeological and Environmental Survey Center of the Xinjiang Uygur Autonomous Region Bureau of Geology.

## 4. Results and analysis

### 4.1. Ground temperature measurement results

(1) Land surface temperature inversion results

**Fig 5:**
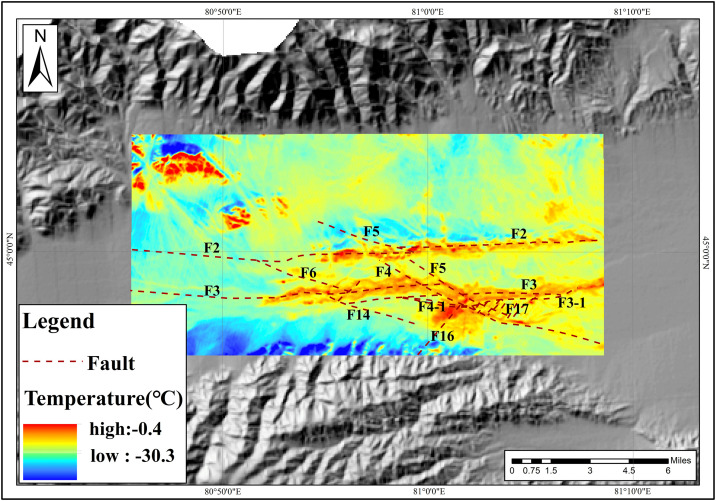
Land surface temperature inversion for winter 2021.

**Fig 6:**
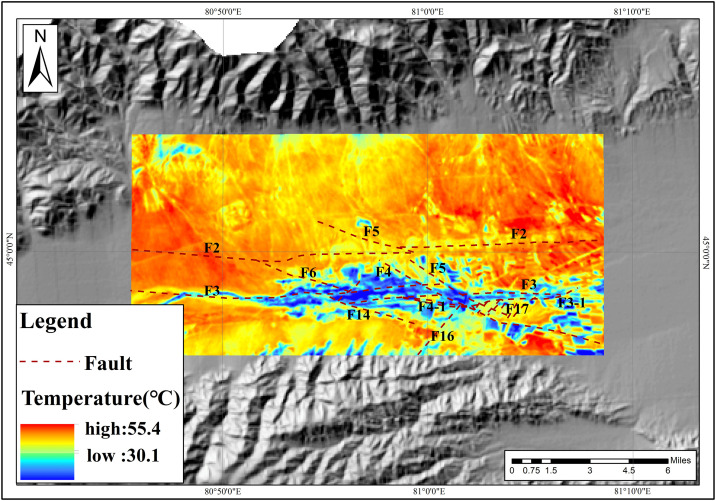
Land surface temperature inversion for summer 2021.

**Fig 7:**
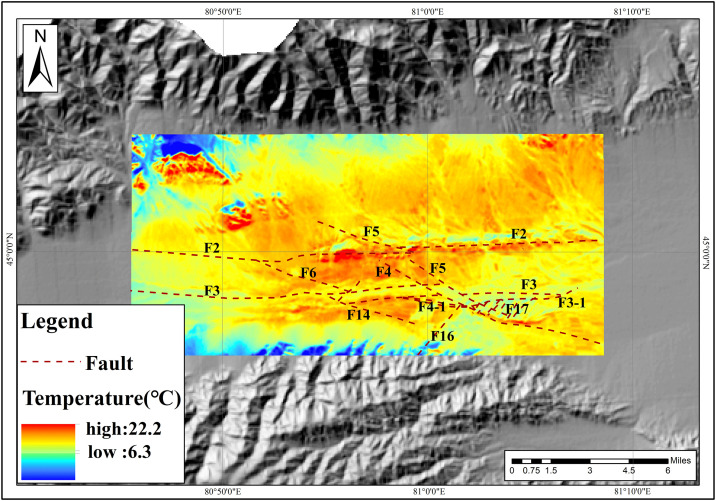
Land surface temperature inversion for winter 2022.

**Fig 8:**
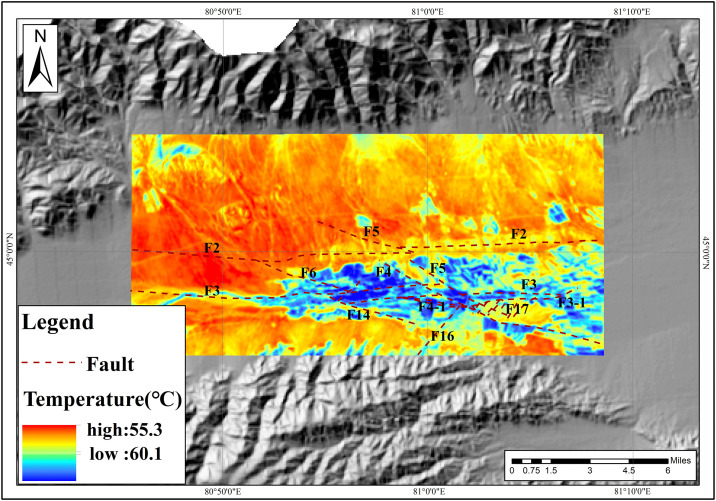
Land surface temperature inversion for summer 2022.

In this study, Landsat-8 remote sensing data with low cloud cover during daytime and nighttime hours from 2021 to 2022 were selected from a geospatial data repository for land surface temperature retrieval. The data selection criteria were as follows: (1) Cloud cover less than 10% to ensure image quality. (2) Time coverage included June (summer) and December (winter) to compare seasonal variations in surface temperature. The results are shown in Figs 5–8.

**Fig 9:**
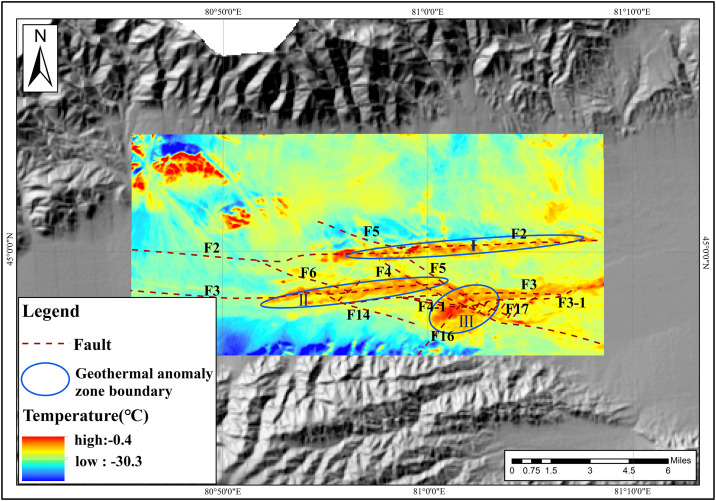
Land surface temperature inversion results of the study area.

As shown in Figs 5–8, the temperature distribution in the study area exhibits significant seasonal variations and regional differences. Fig 5 shows the surface temperatures in the study area during the 21 winters, with temperatures ranging from −30.3 ~ −0.4°C; temperatures in the mountainous areas to the north and south are significantly lower than those in the central region, where faults are densely concentrated. Fig 6 shows the surface temperatures in the study area during the 21 summers, with temperatures ranging from 30.1 ~ 55.4°C. with temperatures near the Bortala River in the central region being significantly lower. Fig 7 shows surface temperatures in the study area during the winter of Year 22, with a range of −6.3 ~ 22.2°C; temperatures on the central plain were generally higher. Fig 8 shows surface temperatures in the study area during the summer of Year 22, with a range of 26.1 ~ 55.3°C; temperatures near the Bortala River in the central region were lower than those in the mountainous areas on either side. Temperatures are generally lower in winter, with surface temperatures ranging from −30°C ~ 22°C; in summer, temperatures rise markedly, with surface temperatures fluctuating between 26°C and 55°C. Specific spatial characteristics are as follows: the northern and southern edges, due to their higher elevations, are the coldest regions; in the central region, urban settlements and villages along the Bortala River lack vegetation cover, resulting in mostly bare ground. During summer days, direct sunlight causes significant heat absorption, while during winter nights, poor thermal retention leads to high heat loss, resulting in pronounced temperature variations in these areas. Ultimately, land surface temperature retrieval data from the winter of 2021, which exhibited pronounced thermal anomalies, was selected as the basis for this study’s land surface temperature retrieval results, as shown in Fig 9.

Land surface temperature retrieval reveals the presence of multiple temperature anomalies in the study area. A high-temperature anomaly was observed in the northwest corner; however, since this area is exposed to light-colored granite, which strongly absorbs solar heat, it is inferred to be a false anomaly of non-geothermal origin. The three temperature anomaly zones in the central part are clearly distributed linearly along the F2, F3, F3-1, and F17 fractures. Consequently, three geothermal anomaly areas with areas of 19.46 km^2^, 15.88 km^2^, and 15.55 km^2^, respectively, were ultimately delineated in the central region. Thus, it can be seen that the geothermal fields in the study area are significantly influenced by tectonic factors, and it is inferred that F2, F3, F4, F4-1, F3-1, and F17 are the primary heat-controlling structures in the region.

(2) Ground temperature measurement results

In this study, temperatures were measured using electronic thermometers at a total of 63 water wells within the study area, combined with logging data from two geothermal wells. The horizontal and vertical characteristics of the geothermal field in the study area are analyzed as follows:

a. Horizontal geothermal field characteristics

Based on actual temperature measurements from water wells in the study area, the measured temperature data are presented in Table 1.

**Table 1:** Shallow ground temperature measurement data.

Number	Ground elevation(m)	Temperature (°C)	Buried depth(m)	Water level(m)	Number	Ground elevation(m)	Temperature (°C)	Buried depth(m)	Water level(m)
J01	1325.68	12.0	2.90	1322.78	J34	1319.00	12.3	1.15	1317.85
J02	1343.03	12.0	1.80	1341.23	J35	1318.00	40.0	—	—
J03	1374.45	12.0	3.30	1371.15	J36	1352.00	42.0	—	—
J04	1385.48	12.0	4.20	1381.28	J37	1345.00	10.0	—	—
J05	1364.00	8.4	13.50	1350.50	J38	1382.00	10.8	—	—
J06	1360.47	10.0	6.60	1353.87	J40	1375.00	16.7	—	—
J07	1359.18	10.0	14.84	1344.34	J41	1345.00	9.2	11.20	1333.80
J08	1352.00	9.0	9.60	1342.40	J42	1340.00	8.7	6.70	1333.30
J09	1346.00	8.0	5.50	1340.50	J43	1316.00	8.5	5.40	1310.60
J10	1335.59	7.3	4.00	1328.34	J44	1377.00	10.4	33.03	1343.97
J11	1316.36	11.0	1.20	1315.16	J45	1396.00	10.5	55.01	1340.99
J12	1318.00	8.6	1.70	1316.30	J46	1351.00	6.9	—	—
J13	1322.18	11.0	3.80	1318.38	J47	1365.00	9.8	15.17	1349.83
J14	1313.00	6.8	2.17	1310.83	J48	1350.00	9.8	6.20	1343.80
J15	1316.00	7.5	4.54	1311.46	J49	1332.00	7.1	13.61	1332.00
J16	1314.35	7.0	3.40	1310.95	J50	1365.90	10.0	18.00	1347.90
J17	1305.00	6.9	3.40	1301.60	J51	1359.00	11.9	10.83	1348.17
J18	1314.00	9.6	9.40	1304.60	J52	1348.00	10.2	—	—
J19	1299.48	8.5	1.10	1298.38	J53	1271.00	10.6	2.15	1268.85
J20	1298.00	9.5	6.05	1291.95	J54	1213.00	7.8	9.00	1204.00
J21	1280.00	9.3	4.45	1275.55	J55	1222.00	9.3	12.00	1210.00
J23	1268.00	9.2	4.97	1263.03	J56	1216.00	11.5	90.00	1126.00
J24	1240.00	10.0	—	—	J57	1288.00	14.9	125.30	1162.70
J25	1237.84	9.3	4.40	1233.44	J58	1224.00	7.1	3.15	1220.85
J26	1370.81	9.8	40.00	1330.81	J59	1374.00	6.1	3.34	1370.66
J27	1316.00	34.0	0.25	1315.75	J60	1443.00	7.6	6.57	1436.43
J28	1322.04	39.0	1.90	1320.14	J61	1420.00	9.6	70.00	1350.00
J29	1309.00	8.3	1.45	1307.55	J62	1482.00	9.8	3.10	1478.90
J30	1332.54	14.0	4.00	1328.54	J63	1316.00	7.4	2.51	1313.49
J31	1338.00	17.1	4.01	1333.99	J57	1288.00	14.9	125.30	1162.70
J32	1325.00	10.5	1.58	1323.42	J64	1441.00	8.4	10.00	1431.00
J33	1320.00	22.6	0.98	1319.02					

As shown in Table 1, there are significant differences in well temperatures across different tectonic zones within the study area: in the northern part, on the hanging wall of the F2 fault, the temperatures of wells J62, J61, J44, and J45 range from 9.6 ~ 10.5°C; wells J03 and J04 at the intersection of the F4-1 and F3-1 faults recorded temperatures of 12°C; in the southern foothills, wells J52, J50, J48, J47, and J07 in the intersection zone of the F16 and F12 faults range from 9.8 ~ 10.2°C, with temperatures on the eastern side of the F16 fault significantly higher than those on the western side; the temperature of well J53 in the intersection zone of the F3-1 and F17 faults in the eastern section of the central Shengshan area is 10.6°C, which is higher than the average temperature of surface water and cold springs in the region, indicating a certain thermal anomaly. Overall, the areas surrounding wells J40 and J57 in the southern foothills and the well sections in the Shengshan area show significantly higher temperatures, constituting typical thermal anomaly zones. A comprehensive analysis of the shallow horizontal temperature field in the study area reveals that temperatures are significantly higher than in the surrounding areas on the hanging walls of the F2, F4-1, F3-1, and F17 fault groups and at their intersection zones. This indicates that the F2, F4-1, F3-1, and F17 fault groups possess thermal conductivity and play a key role in the formation of the geothermal field.

**Fig 10:**
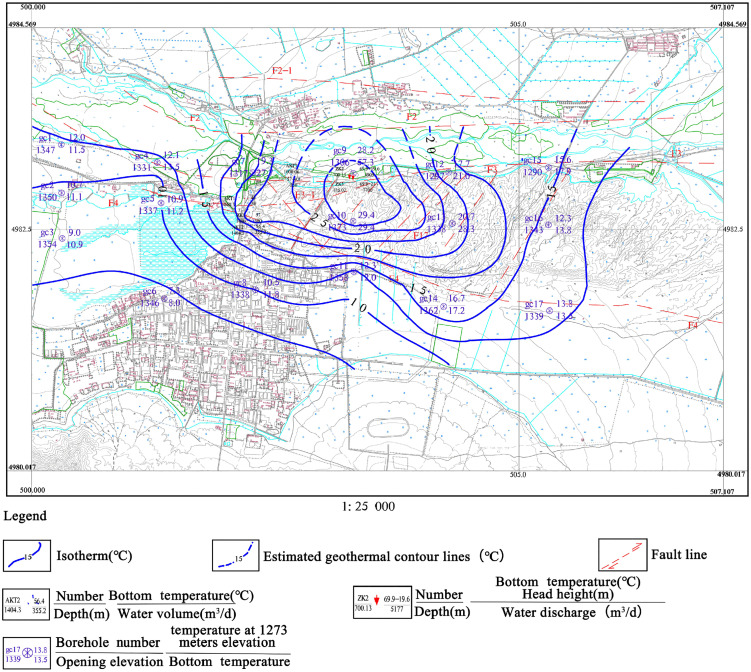
Geothermal isotherm map at 1273 m elevation in the study area. The figure shows the 1273 m elevation geothermal isotherms derived from temperature measurement data and the inferred geothermal isotherms within the Sacred Mountain area of the study region (i.e., the area between F2-1 and F4 in Fig 2).

To investigate the deep-seated characteristics of the planar temperature field, a geothermal isotherm map at an elevation of 1273 m was compiled using temperature measurement data from geothermal and water wells (as shown in Fig 10).

The contour maps show that the highest surface temperatures are located near boreholes ZK2 and ZK3. Overall, temperatures exhibit a trend of gradually decreasing outward from the Shengshan area—situated between the F3-1 and F4 fractures—in a ripple-like pattern. However, the rate of decrease exhibits a certain directional variation, with the major axis oriented roughly east-west, consistent with the location and extension direction of the fault structures. This indicates that the geothermal field is directly controlled by tectonic uplift. With well-developed rock fractures in the shallow zone and frequent circulation and exchange of geothermal water, the shallow geothermal field is influenced to some extent by the deep geothermal field, causing the extension direction of the geothermal field to generally align with the direction of the tectonic lines.

b. Characteristics of the Vertical Geothermal Field

**Fig 11:**
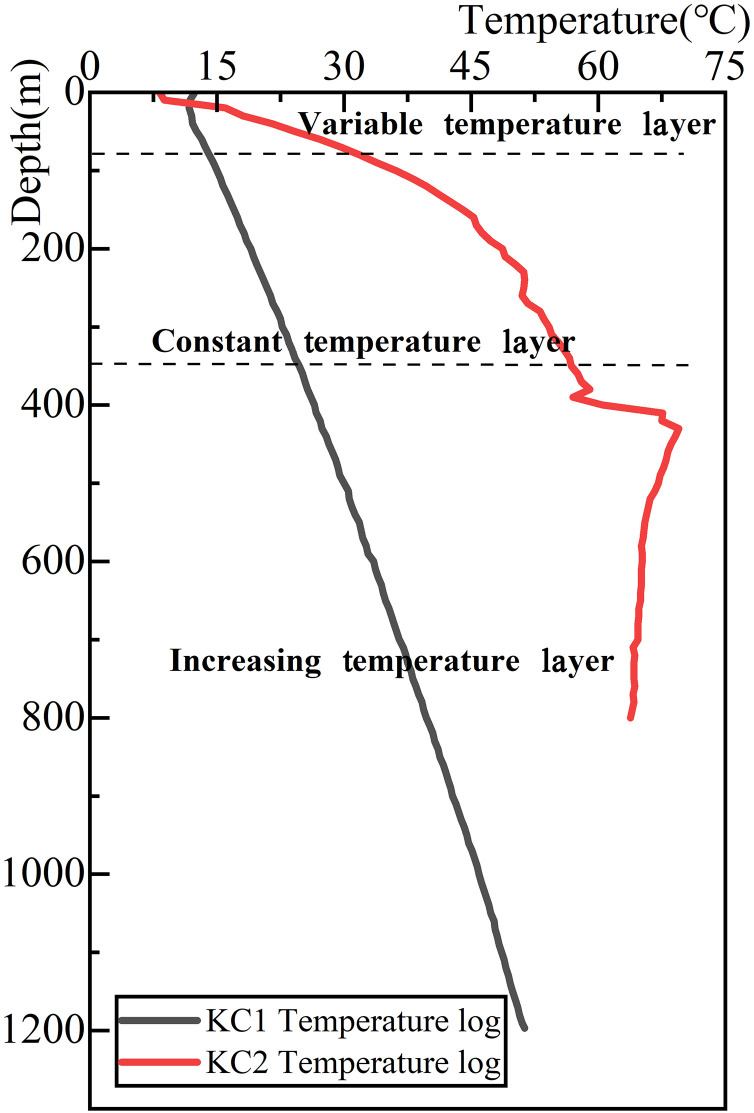
Geothermal well temperature measurement curves.

**Fig 12:**
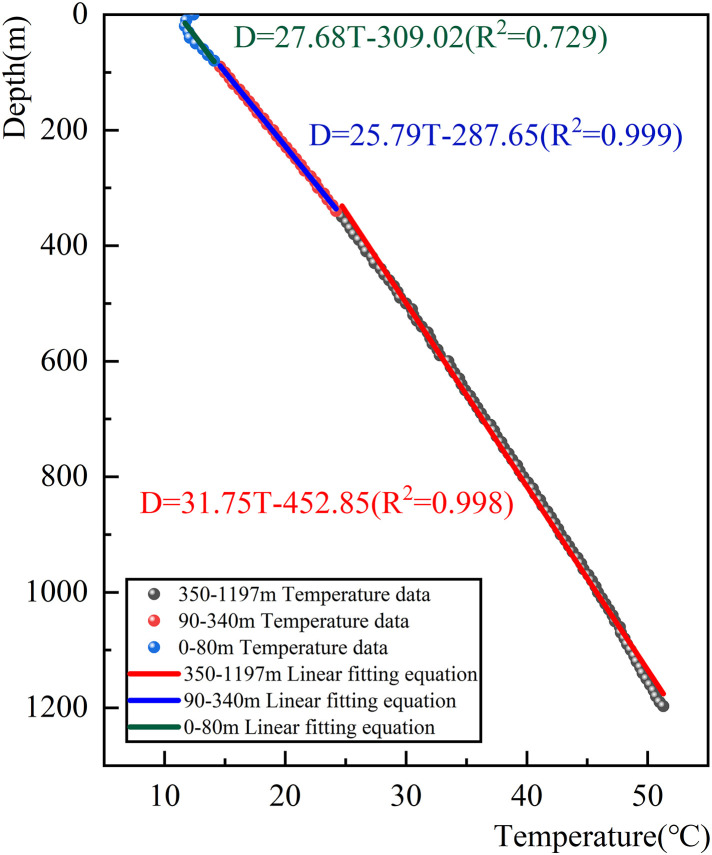
Well KC1 temperature curve.

**Fig 13:**
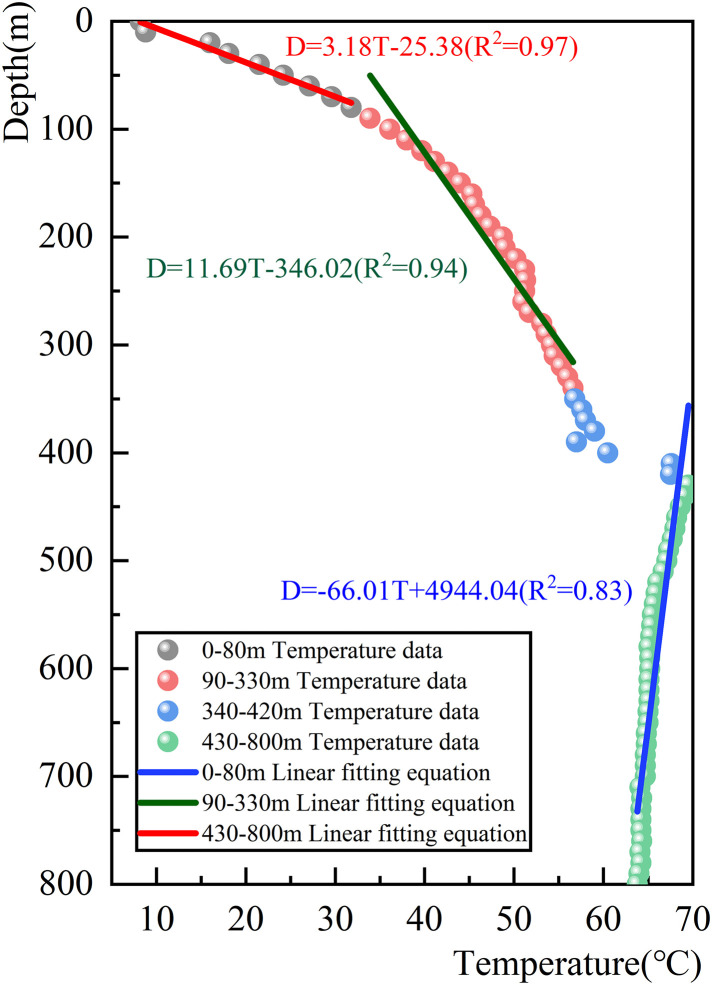
Well KC2 temperature curve.

To investigate the distribution patterns of the vertical geothermal field, temperature measurement curves for geothermal wells KC1 and KC2 in the study area were plotted based on actual temperature measurements, as shown in Figs 11–13.

The temperature curves from the geothermal wells show that temperatures in both wells increase steadily with depth. To thoroughly analyze the vertical characteristics of the geothermal field, a segmented linear regression analysis was performed on the temperature curves. In the 0 ~ 80 m interval, the temperature rises rapidly. The results of the segmented linear regression analysis show that the geothermal gradient for Well KC1 is 2.1°C/100 m (R^2^ = 0.729), while that for Well KC2 is 29.5°C/100 m (R^2^ = 0.97). The linear relationship for Well KC2 is highly significant, whereas the linear relationship between temperature and depth for Well KC1 is relatively weak. This stratum is significantly influenced by surface climate and belongs to the variable temperature zone; 80 ~ 350 m interval: The rate of increase in stratigraphic temperature slows. The geothermal gradient for borehole KC1 is 3.9 °C/100 m (R^2^ = 0.999), and for borehole KC2, it is 9.3 °C/100 m (R^2^ = 0.94). Both exhibit a significant linear relationship, with temperature changes tending to level off, corresponding to the development of the constant temperature zone; 350 ~ 1200 m interval: The rate of temperature increase accelerates again. The geothermal gradient in Well KC1 is 3.1°C/100 m (R^2^ = 0.998), and in Well KC2 it is 1.6°C/100 m (R^2^ = 0.83), reflecting the enhanced influence of deep heat sources, corresponding to the temperature-increasing zone. In Well KC2, a sudden temperature jump from 56.6°C ~ 69.5°C was observed in the 340 ~ 430 m interval, indicating that this section exposed the central region of a thermally conductive structure, characterized by fractured rock and high thermal conductivity; in the 430 ~ 800 m interval, the slope of the equation is negative, and the temperature gradually decreases to 63.8°C, indicating that the borehole has penetrated the thermally conductive structure, resulting in a temperature drop.

Combined with an analysis of regional groundwater circulation conditions, the aforementioned temperature anomalies are closely related to fault-controlled water and heat conduction structures. The temperature jump observed in Borehole KC2 between 399 and 424 m indicates that the borehole traversed a fractured zone where east-west and northeast-southwest faults intersect. On the one hand, the fault intersection zone provides an efficient pathway for deep thermal fluids to migrate upward, creating local thermal anomalies; on the other hand, the fractured zone along the fault intersection enhances the efficiency of heat exchange between groundwater and the surrounding rock, resulting in non-uniformity in the vertical temperature gradient. It should be noted that the formation of the geothermal field in the study area may not be controlled solely by the combination of east-west and northeast-southwest faults; other potential influencing factors may include differences in the thermophysical properties of rocks and the distribution of deep heat sources.

The results of the above quantitative analysis collectively indicate that the Wenquan County geothermal field belongs to a typical convective geothermal system.

**Fig 14:**
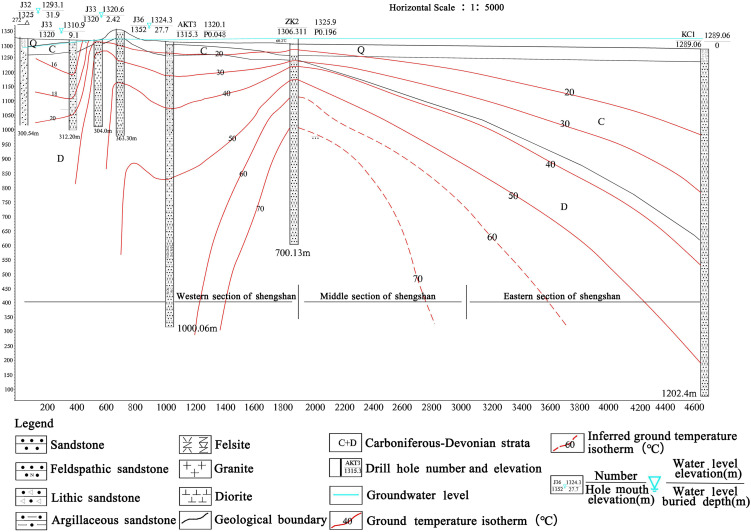
Vertical geothermal temperature profile. Vertical geothermal profiles along an east-west direction through seven wells, including KC1, ZK2, and J32 (well locations are shown in Fig 2). Geothermal isotherms are spaced at 10°C intervals, and dashed lines indicate inferred geothermal isotherms. The vertical axis is in meters above sea level, and the horizontal axis is in meters of east-west distance.

To investigate the vertical variation patterns of the geothermal field, a geothermal profile was plotted along the east-west direction of the field, as shown in Fig 14.

As shown in Fig 14, the vertical geothermal isotherms in the western and central sections of Shengshan form two upward bulges centered around the ZK2 borehole area: the first bulge is located near J36 in the western section of Shengshan and extends in the direction of the granite intrusion, indicating that geothermal fluids are rising from the basement along the fractured zone created by the magmatic intrusion; the second bulge is located in the ZK2 area of the central section of Shengshan, where geothermal isotherms are densely concentrated and the geothermal gradient is significantly higher. High-temperature hot water rapidly rises in this area. This phenomenon indicates that the ZK2 area is controlled by the F17 fault group, which strikes in a northeast-southwest direction. The steep dip of these faults and their connection to the basement accelerate the migration of geothermal fluids. It is inferred that this area serves as a conduit for the upward flow of terrestrial heat flow, reaching a depth of up to 2.05 km.

(3) Comprehensive comparison

A comprehensive comparison of the results from remote sensing-based land surface temperature retrieval and field temperature measurements reveals that the temperature anomaly zones generally coincide with the distribution of the geothermal field, concentrating in the intersection area of the F4 fault and the F3-1 and F17 faults. These findings mutually validate each other’s authenticity. At the same time, they demonstrate the controlling role of faults such as F4, F3-1, and F17 in the formation of the geothermal field.

### 4.2. Terrestrial heat flow and rock radiogenic heat production rate

Since only the heat transferred during steady-state heat conduction can characterize the heat flux, we selected data from the temperature-depth curves of AKT1, AKT2, and AKT3, where temperature and depth exhibit good linearity, to calculate the geothermal gradient. Using the well-fitted (R^2^ > 0.9) sections from geothermal wells KC1 and KC2 mentioned earlier, the average geothermal gradient of the study area was calculated as 11.53 °C/100 m. Combined with the measured average rock thermal conductivity of 2.776 W/(m·°C) (see Table 2), the calculated terrestrial heat flow value for the Wenquan County geothermal field is 320 mW/m^2^, which is significantly higher than the average terrestrial heat flow value of 40 ~ 45 mW/m^2^ at the northwestern margin of the Junggar Basin in Xinjiang, indicating that Wenquan County possesses a relatively favorable terrestrial heat flow background. The formation of this high heat flow background is closely related to factors such as the heat transfer pathways provided by deep-large fault zones, heat generated by the decay of radioactive elements in the crust, and enhanced heat transfer due to groundwater convection. This reflects that the study area possesses a sound thermodynamic basis for the formation and accumulation of geothermal resources.

**Table 2:** Statistical table of thermal conductivity of various rocks.

Geological Time	Rock Thermal Conductivity (w/(m·°C))	Mean Value of Rock Thermal Conductivity (w/(m·°C))
Cambrian System	2.291 ~ 2.783	2.521
Ordovician System	2.112 ~ 3.015	2.742
Devonian System	2.186 ~ 3.884	2.978
Carboniferous System	1.786 ~ 4.31	2.863

To investigate the impact of radiogenic heat production in rocks within the study area on the geothermal field, we conducted tests to measure the radioactive content of various rock types and analyzed the concentrations of U, Th, and K, as well as calculated the radiogenic heat production rates. The results are shown in Table 3.

**Table 3:** Calculated radiogenic heat production rates of various rock types.

Rock Type		Density (kg/m^3^)		U (ug/g)		Th (ug/g)		K (%)		Heat Production Rate (μw/m^3^)	Average Heat Production Rate (μw/m^3^)
		Interval Value	Mean Value	Interval Value	Mean Value	Interval Value	Mean Value	Interval Value	Mean Value		
Igneous Rock	Fine-grained Diorite	2740 ~ 2860	2800	0.4 ~ 0.9	0.65	8.43	8.43	0.84 ~ 2.01	1.425	0.92	1.262
	Felsite	2700 ~ 2870	2785	4.3 ~ 12.2	8.25	9.83 ~ 6.31	8.07	2.07 ~ 2.97	2.52	3.01	
	Altered Basalt	2880	2880	1.2	1.2	—	—	1.56	1.56	0.49	
	Hornblende Macadam	2670	2670	1.57	1.57	1.14	1.14	1.63	1.63	0.63	
Metamorphic Rock	Leptynite	2710 ~ 2780	2737	1.1 ~ 4.1	2.133	5.5 ~ 9.07	7.285	1.09 ~ 2.38	1.653	1.22	1.774
	Quartz Schist	2770 ~ 2780	2775	1.45 ~ 9.71	5.58	7.14 ~ 10.7	8.92	2.1 ~ 2.59	2.345	2.33	
	Metamorphic Siltstone-Mudstone	2540 ~ 2770	2666	0.6 ~ 6.77	2.842	1.74 ~ 9.86	6.192	2.56 ~ 3.78	14.9	2.53	
	Schistose Quartzite	2510	2510	7.01	7.01	8.27	8.27	3.20	3.20	2.49	
	Felsic Hornfels	2640	2640	0.7	0.7	—	—	1.37	1.37	0.30	
Sedimentary Rock	Lithic Sandstone	2580 ~ 2770	2647	1.16 ~ 5.568	2.802	1.2-10.73	5.491	0.99 ~ 2.88	1.721	1.24	2.934
	Lithic Arkose	2570 ~ 2670	2622	1.9 ~ 97.2	23.43	3.24 ~ 21.3	9.003	0.296 ~ 2.83	1.815	6.62	
	Crystal-vitric Tuff	1850 ~ 2720	2585	2.73 ~ 74.5	13.115	2.33 ~ 21.72	6.646	0.72 ~ 3.34	2.263	3.87	
	Metamorphic Argillaceous Siltstone	2590 ~ 2670	2633	2.72 ~ 4.51	3.694	2.63 ~ 3.95	3.226	0.769 ~ 2.8	1.986	1.33	
	Quartz Sandstone	2770	2770	3.66	3.66	7.1	7.1	1.45	1.45	1.61	

Table 3 shows that there are differences in the rock radiogenic heat production rates among different lithologies in the study area: The average rock radiogenic heat production rate of igneous rocks in the study area is 1.262 μW/m^3^. Among these, the radiogenic heat production rate of felsite is 3.01 μW/m^3^, which is higher than the global average radiogenic heat production rate of granite (2.5 μW/m^3^). This indicates that felsite veins make a significant contribution to the terrestrial heat flow in the study area; Metamorphic rocks are primarily composed of granulites and meta-silty mudstones, formed mainly by hydrothermal alteration under medium- to high-temperature conditions, with an average radiogenic heat production rate of 1.774 μW/m^3^; The average radiogenic heat production rate of sedimentary rocks is 2.934 μW/m^3^, which is higher than that of typical granite. This is likely due to the enrichment of radioactive elements in the hanging wall of heat-controlling faults, resulting in relatively high radiogenic heat production rates in some sedimentary rocks. Overall, sedimentary rocks contribute the most to the terrestrial heat flow through radiogenic heat production; among igneous rocks, only felsite makes a significant contribution, while the others contribute little. The average radiogenic heat production rate for the three radioactive elements in the study area is 1.99 μW/m^3^, which translates to a contribution of 1.99 mW/m^2^ per kilometer of thickness to the terrestrial heat flow. While this represents a certain contribution to the terrestrial heat flow, the magnitude is relatively small. It can thus be concluded that radiogenic heat production serves as a stable heat source for the geothermal field, but it is not the primary heat source.

### 4.3. Geophysical exploration and drilling results

A total of eight CSAMT profiles (N1 ~ N8) were completed during this study. The geophysical exploration and drilling results for the study area are shown in Figs 15–22.

The geophysical profiles reveal that there is one low-resistivity anomaly zone on Survey Line N1, with its center at Point 61 and a buried depth of 1130 m; there are two low-resistivity anomaly zones on Survey Line N2, with their centers at Points 31 and 81, at buried depths of 420 m and 530 m, respectively; and there is one low-resistivity anomaly zone on Survey Line N3, with its center at Point 31 and a buried depth of 1230 m; Survey Line N4 exhibits one low-resistivity anomaly zone, with its center at Point 136 and a buried depth of 1855 m; Survey Line N5 exhibits one low-resistivity anomaly, with its center at Point 144 and a buried depth of 1222 m; Survey Line N8 exhibits one low-resistivity anomaly, with its center at Point 9 and a buried depth of 1121 m; Survey Lines N6 and N7 show no low-resistivity anomaly zones. Based on the low-resistivity distribution characteristics of the eight geophysical survey line profiles, it is inferred that the aforementioned low-resistivity anomalies were caused by the fracturing and water-filling of high-resistivity host rock under faulting. These correspond to the locations of the F2, F2-1, F4, and F5 fractures, which generally align with the locations of surface outcrops and areas of land surface temperature retrieval anomalies. Additionally, it can be concluded that the rock mass at these locations is fractured, has high porosity, and possesses strong permeability and water-conducting capacity.The RQD values from the drilling results of the AKT1, AKT2, and AKT3 geothermal wells are plotted in bar charts, as shown in Fig 23, Fig 24, and Fig 25.

**Fig 15:**
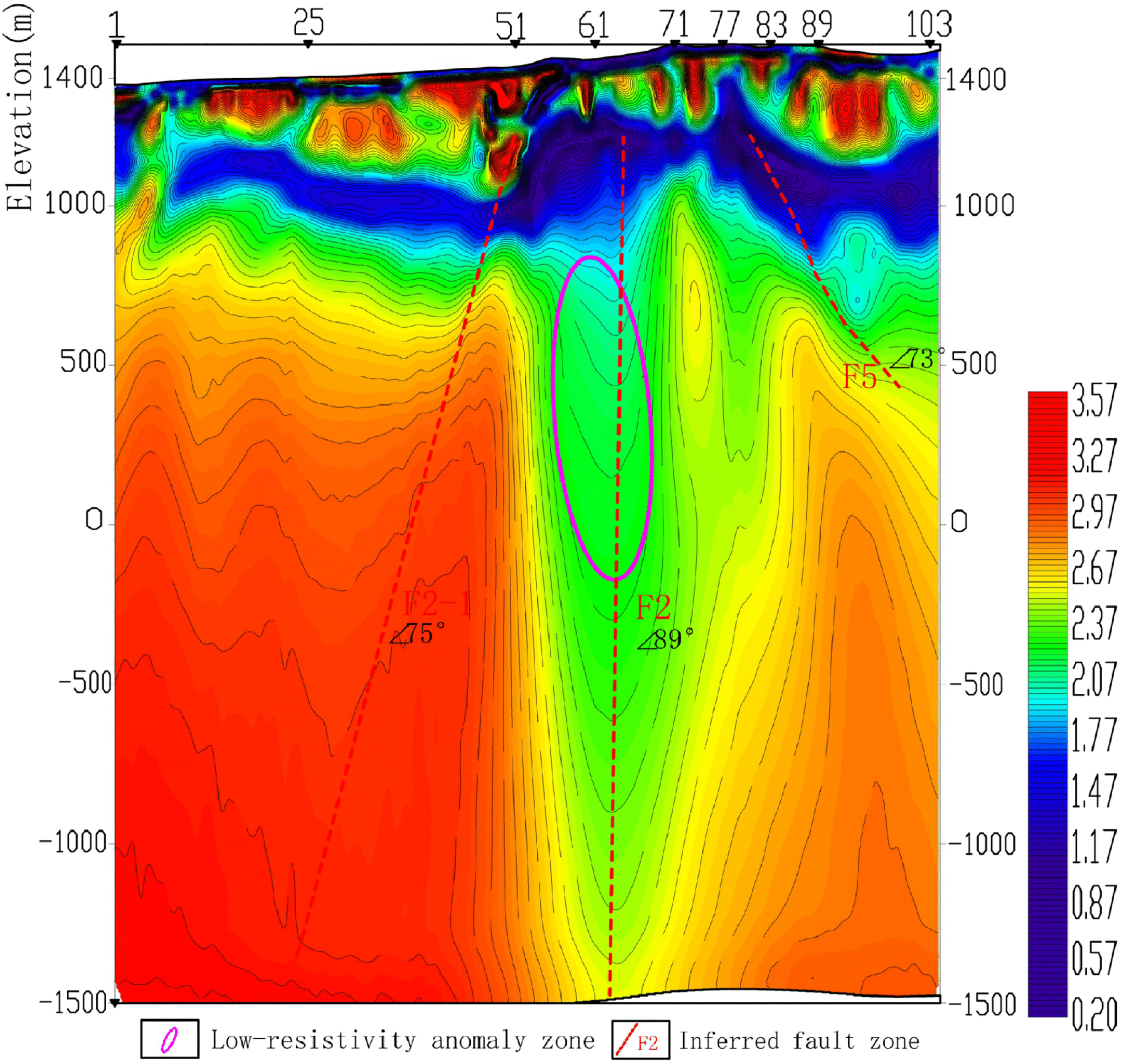
N1 apparent resistivity contour map.

**Fig 16:**
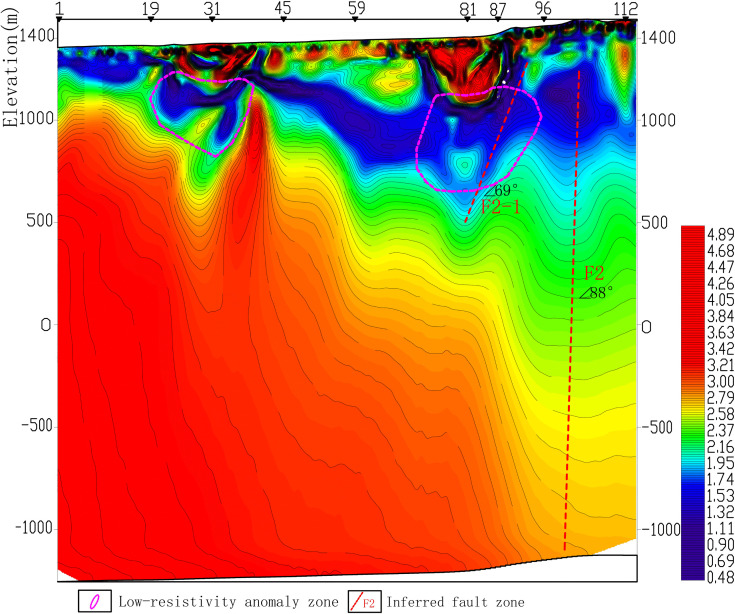
N2 apparent resistivity contour map.

**Fig 17:**
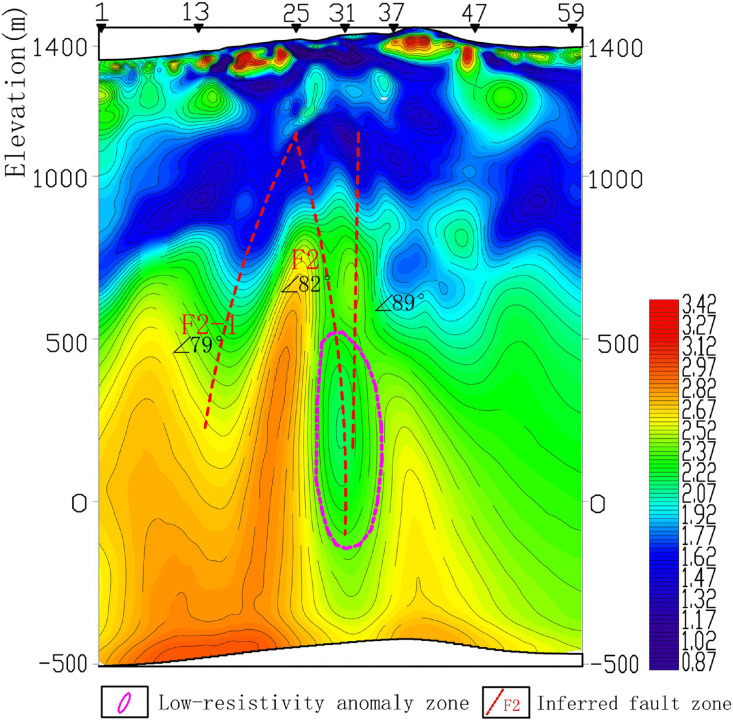
N3 apparent resistivity contour map.

**Fig 18:**
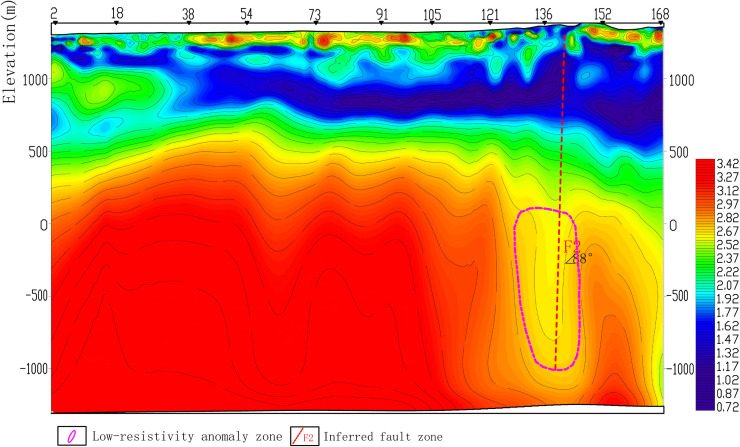
N4 apparent resistivity contour map.

**Fig 19:**
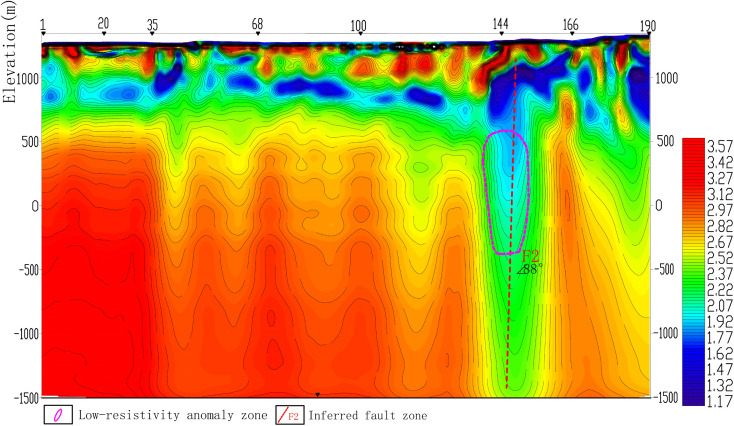
N5 apparent resistivity contour map.

**Fig 20:**
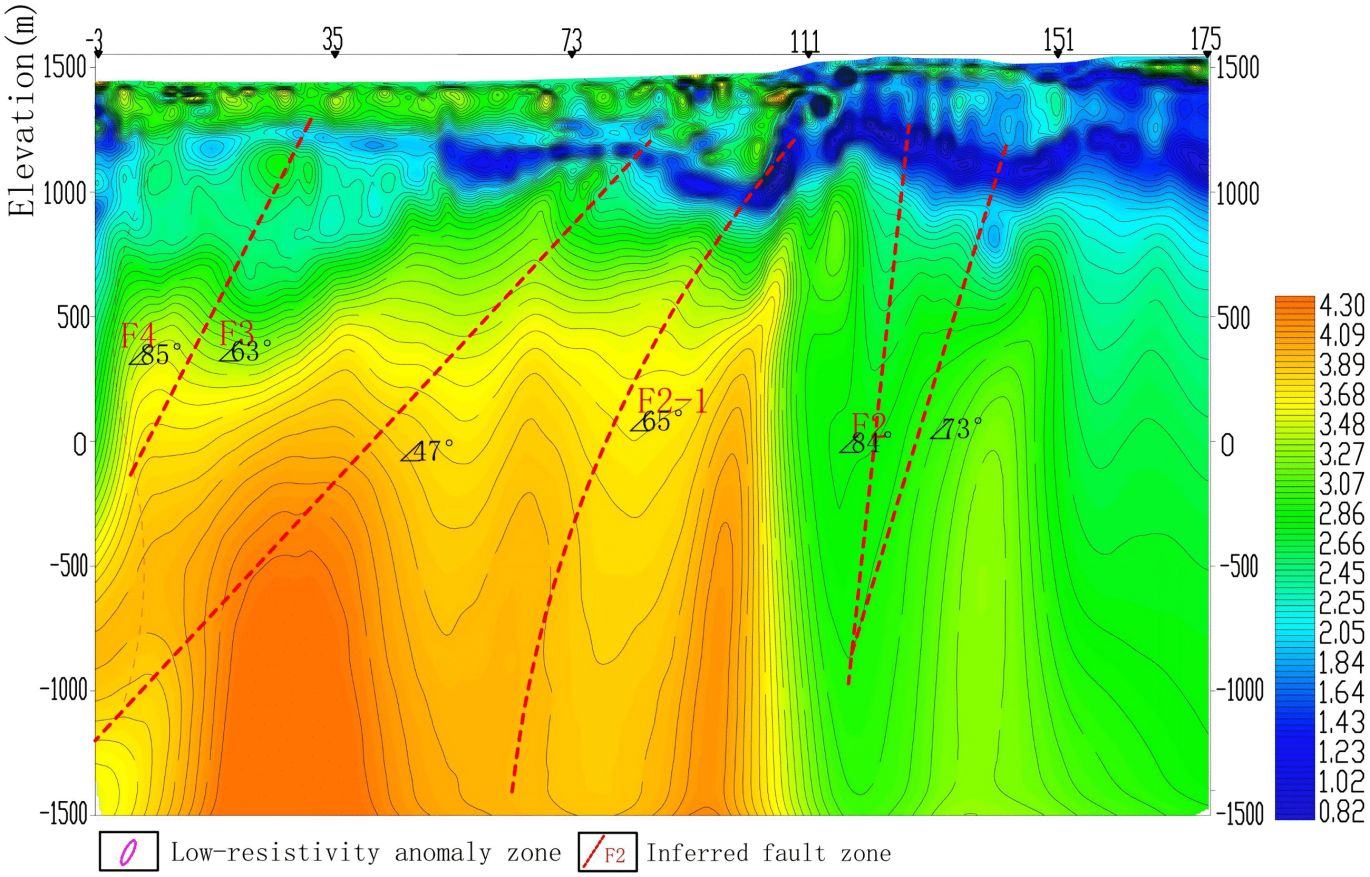
N6 apparent resistivity contour map.

**Fig 21:**
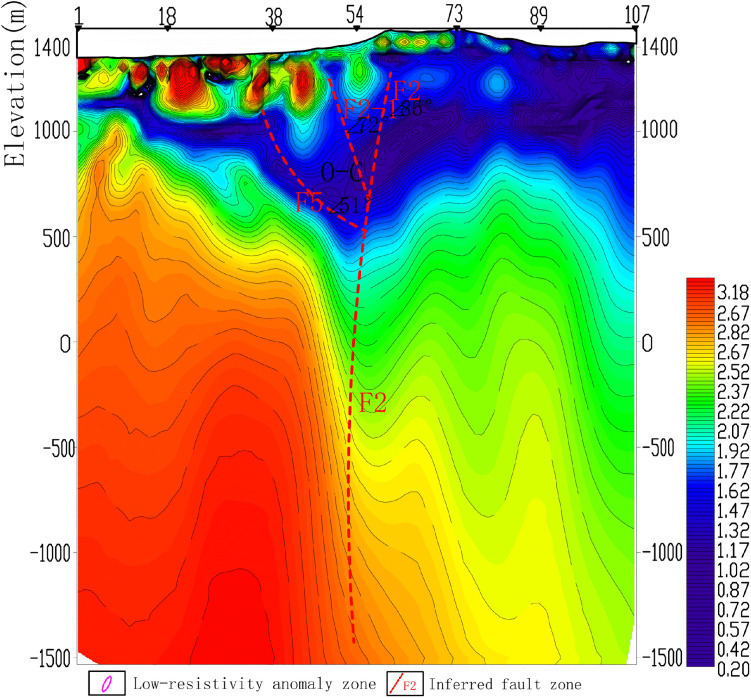
N7 apparent resistivity contour map.

**Fig 22:**
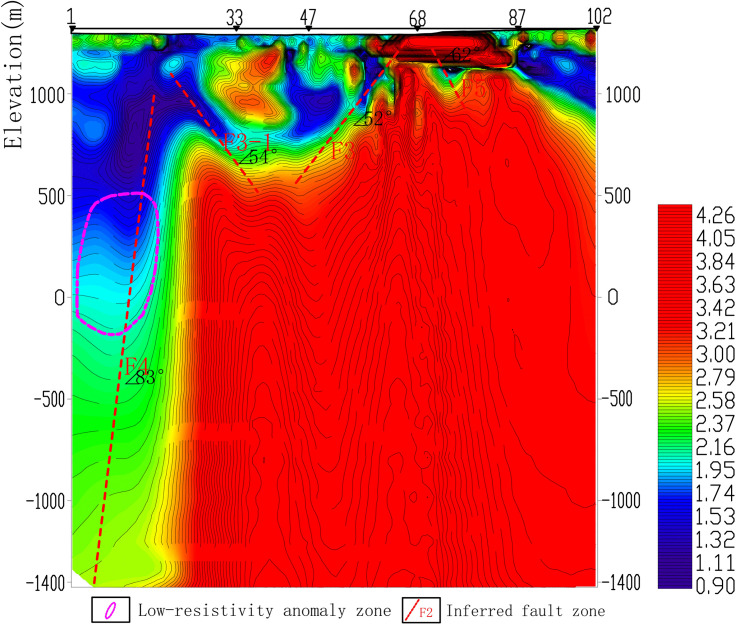
N8 apparent resistivity contour map.

**Fig 23:**
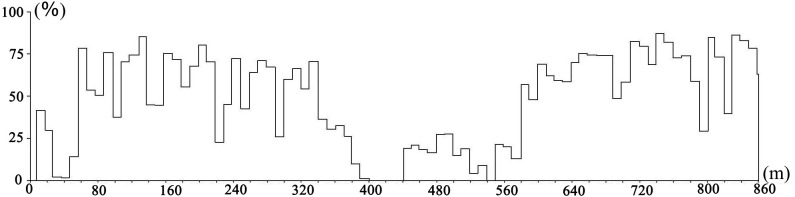
RQD histogram of borehole AKT1.

**Fig 24:**
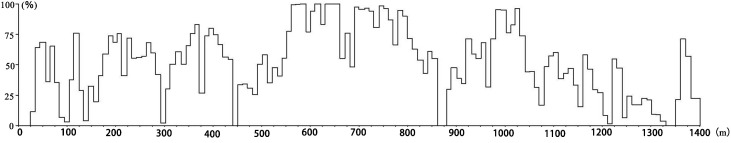
RQD histogram of borehole AKT2.

**Fig 25:**
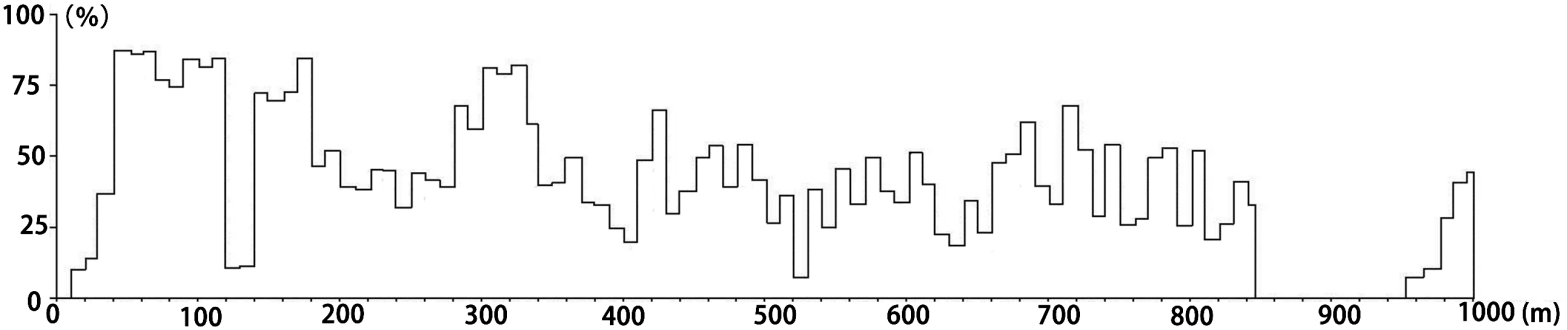
RQD histogram of borehole AKT3.

The RQD histogram for the geothermal wells shows that: in the core samples recovered from the 360 ~ 580 m section of Well AKT1, RQD values mostly range from 0 ~ 50, and the core appears fragmented; in the core samples recovered from the 845 ~ 980 m section of Well AKT3, the RQD value is 0, and the core appears powdered. Based on the principle that lower RQD values indicate poorer rock quality and reduced rock mass integrity, it is determined that Well AKT1 and Well AKT3 exposed Fault F3-1 at depths of 360 ~ 580 m and 845 ~ 980 m, respectively, which is consistent with the geophysical interpretation of the F3-1 fault fracture zone. The RQD values of the AKT2 well core at depths of 440 ~ 550 m, 800 ~ 950 m, and 1038 ~ 1360 m mostly ranged between 0 ~ 50, which generally coincides with the geophysical inference of the F4 fault location. Additionally, during the exposure of the F3-1 fault in Well AKT3, water inflow significantly increased at depths of 576 ~ 586 m and 419 ~ 475 m, and abrupt changes appeared in the temperature measurement curves, suggesting that F3-1 is a water-conducting fault.

**Fig 26:**
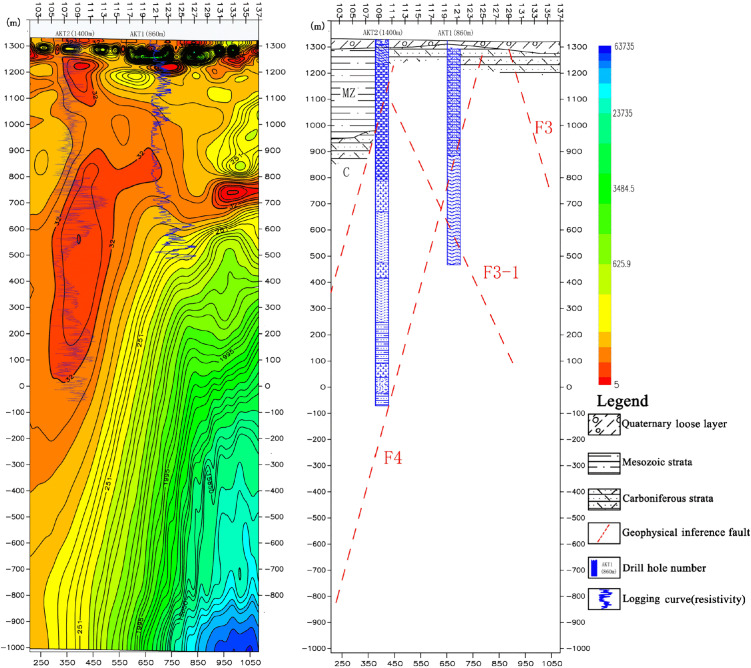
Comparison of geophysical profile and borehole data.

The drilling results from Wells AKT1 and AKT2 were compared with previous studies of Profile M2, as shown in Fig 26.

A comparison of the geophysical profiles shows that the variations in the AKT1 and AKT2 logging curves are essentially consistent with those of the CSAMT resistivity contours, thereby validating the reliability of the geophysical results.

### 4.4. Results of geothermometer calculations

**Fig 27:**
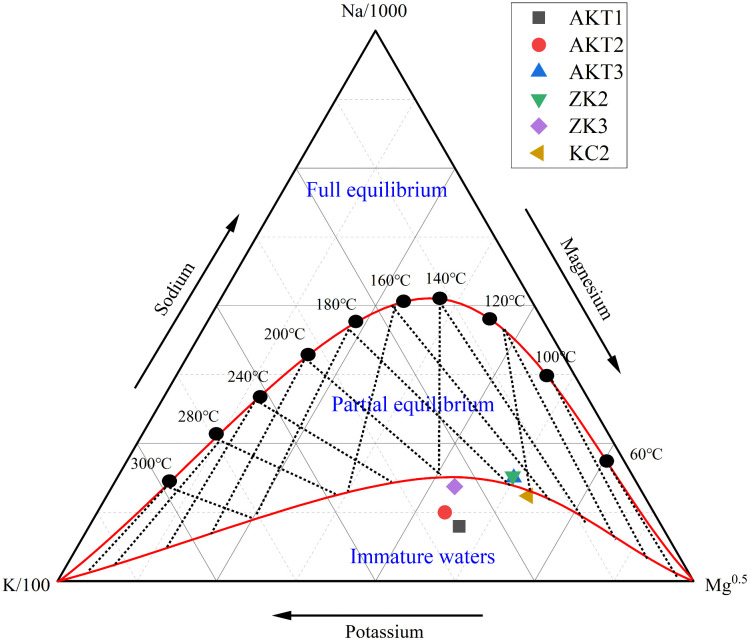
Na-K-Mg ternary diagram.

To investigate the temperature of the geothermal reservoir, this study measured the concentrations of elements used for geothermometer calculations in six geothermal wells (AKT1, AKT2, AKT3, ZK2, ZK3, and KC2) and calculated the corresponding reservoir temperatures. First, water samples from the six groups of geothermal wells in the study area were plotted on a Na-K-Mg triangular diagram, as shown in Fig 27.

As shown in Fig 15, the AKT1, AKT2, KC2, and ZK3 points are located in the “Immature waters” region, indicating that the water-rock reactions for these samples have not reached equilibrium and that the cationic geothermometer is not applicable. The AKT3 and ZK2 water samples are located in the “Partial equilibrium” region, indicating that the K-Mg system has reached equilibrium while the Na-K system has not; therefore, the K-Mg geothermometer can be used to estimate reservoir temperatures.

Based on the formulas, reservoir temperatures were estimated for water samples from the six geothermal wells; the results are shown in Table 4.

**Table 4:** Table of comprehensive geothermometer calculation results.

Number	Content of Temperature Scale Elements				Geothermal Temperature Scale		Measured Wellhead Temperature (°C)	Measured Bottom-hole Temperature (°C)
	K^+^ (mg/l)	Mg^2+^ (mg/l)	Na^+^ (mg/l)	SiO_2_ (mg/l)	Potassium and Magnesium(°C)	Quartz(°C)		
AKT1	2.9	0.7	170.2	37.85	—	111.7	43	46
AKT2	4.6	1.2	201.5	58.92	—	133.3	50.3	56.4
AKT3	5.5	0.2	182.8	60.00	71.4	134.3	52.8	57
ZK2	5.6	0.2	182.1	69.08	102.0	141.7	61.2	69.9
ZK3	5.6	0.7	175.8	71.31	—	143.4	62.4	69.9
KC2	4.1	<0.3	175.35	67.08	—	140.1	57.4	70.1

A comparison with the measured temperatures from geothermal wells in Table 4 shows that the estimated temperature range based on the silica geothermometer is 111.7 ~ 143.4 °C, with an average of 132.4 °C. Compared with the measured temperatures, the estimated temperatures from the silica geothermometer are significantly higher, indicating that they represent the reservoir temperature rather than the temperature at the borehole surface. It was observed that the bottom-hole temperatures of the two geothermal wells, ZK2 and ZK3, were similar, and the estimated values from the silica geothermometer were also relatively close, indicating good internal consistency in the results. The estimated temperature range from the K-Mg temperature index was 71.4 ~ 102.0°C. The estimated values from the K-Mg thermometer are consistent with the trends of the measured bottom-hole temperatures, indicating that the K-Mg thermometer is well-suited for samples from partially equilibrated zones.

Based on the above analysis, this study adopted the SiO_2_ temperature scale—which is less affected by cold water mixing, exhibits good internal consistency, and provides comprehensive data—as the primary basis for estimating geothermal reservoir temperatures. The estimated range for the geothermal reservoir in the study area is 111.7 ~ 143.4°C, with an average of 132.4°C. This indicates that Wenquan County’s geothermal field belongs to a medium-to-low-temperature geothermal system and possesses significant development potential.

### 4.5. Groundwater chemistry and isotope analysis

To analyze the hydrochemical characteristics of the study area, this study collected data on the basic hydrochemical properties of geothermal water from eight geothermal wells through a combination of field sampling and laboratory testing. The hydrochemical characteristics of the geothermal fluids are presented in Table 5.

**Table 5:** Table of basic hydrochemical characteristics of geothermal fluids.

Number	K^+^ (mg/l)	Na^+^ (mg/l)	Ca^2+^ (mg/l)	Mg^2+^ (mg/l)	Cl^-^ (mg/l)	F^-^ (mg/l)	SO_4_^2-^ (mg/l)	HCO_3_^-^ (mg/l)	CO_3_^2-^(mg/l)	H_2_SiO_3_ (mg/l)	H_3_BO_3_ (mg/l)	TDS (mg/l)	Total Hardness (mg/l)	PH
ZK2	3.9	183.6	12.0	0.0	67.4	14.7	247.8	24.4	0.85	90.7	17.41	656.8	30.0	8.75
KC1	5.8	254.5	32.1	2.4	92.2	13.1	393.8	12.2	0.18	79.4	16.27	904.3	90.1	8.33
ZK3	3.86	172.1	26.35	2.9	148.71	14.26	205.86	56.10	1.65	92.9	11.69	619	36.86	8.6
ZK1	3.29	171.9	13.7	3.0	146.15	14.79	166.98	46.20	0.53	77.7	11.59	532	33.04	8.2
KC2	4.1	175.35	12.45	3.0	60.92	14.82	217.74	19.8	3.20	87.2	12.14	654	37.89	9.36
AKT1	2.9	170.2	12.0	0.7	63.1	13.9	223.8	21.4	2.85	49.2	11.42	573.0	33	9.23
AKT2	4.6	201.5	6.0	1.2	62.0	15.1	259.4	36.6	2.80	76.6	14.65	664.1	20.0	9.05
AKT3	5.5	182.8	8.0	0.2	68.4	14.1	257.4	14.6	0.52	78.0	19.8	644.4	21.0	8.7

**Fig 28:**
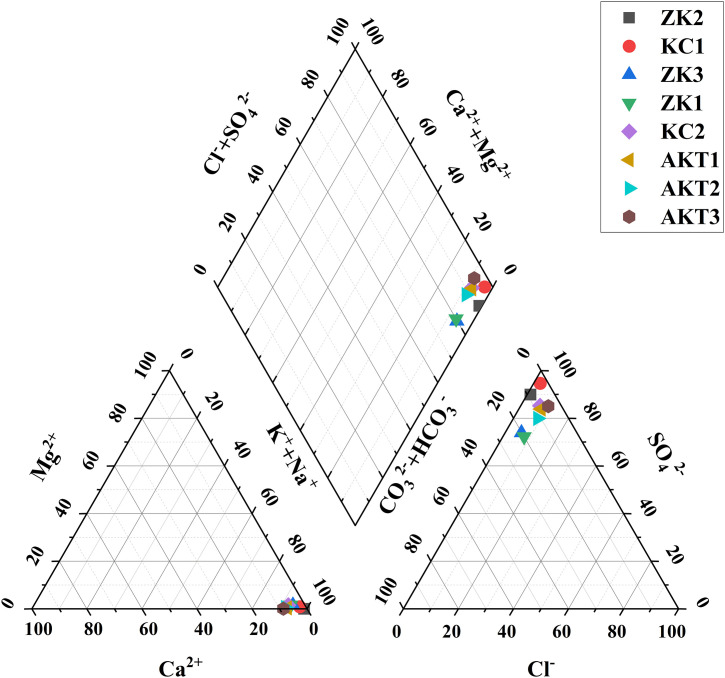
Piper trilinear diagram of geothermal water in the study area.

**Fig 29:**
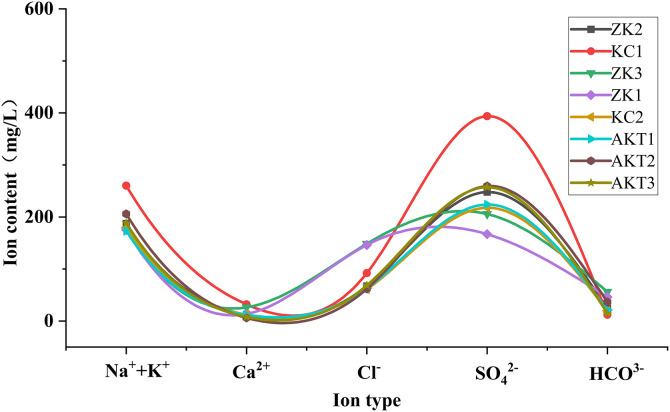
Schoeller diagram of geothermal water in the study area.

Using the hydrochemical classification method of Sergei Alekseevich Shchukarev (based on combinations of anions and cations with milligram equivalent percentages greater than 25%), the geothermal fluids in the study area were all classified as the SO_4_-Na type. According to the Piper trilinear diagram (as shown in Fig 28), the eight water sample groups exhibit a high degree of overlap, indicating significant similarity in their primary ionic composition. This suggests that the geothermal waters in the study area share the same recharge source and hydrogeochemical evolution path. Furthermore, the Schoeller diagram (as shown in Fig 29) reveals relatively high SO_4_^2-^ concentrations and low Ca^2+^ concentrations, indicating that: (a) the aquifer has been subjected to hydrothermal alteration, with large amounts of sulfur and sulfides being oxidized and entering the system as SO_4_^2-^ ions; (b) as the groundwater rises, the decrease in temperature and pressure causes CO_2_ to escape, triggering decarbonation and the formation of CaCO_3_ precipitates, which reduces the Ca^2+^ and HCO_3_^−^ concentrations in the water. This indirectly indicates that the geothermal water in the study area is influenced by tectonic activity and magmatic processes, while the faults provide favorable pathways for the migration of geothermal fluids.

**Fig 30:**
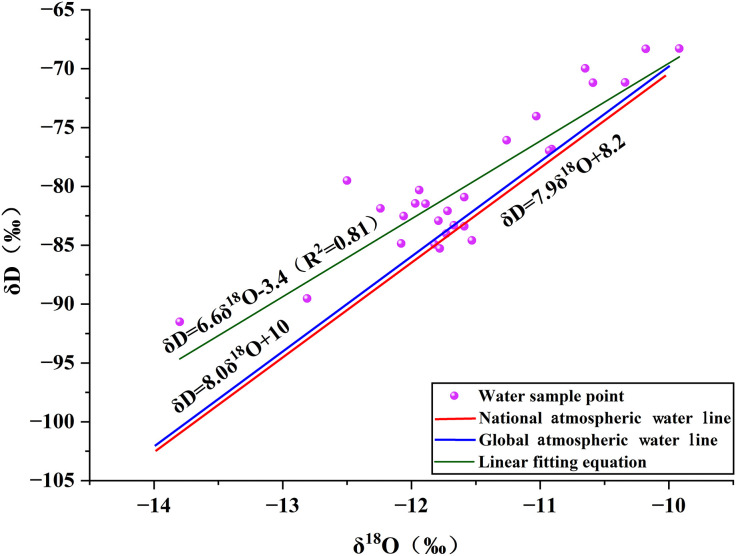
Relationship diagram of δD and δ^18^O.

To analyze the sources and recharge elevations of groundwater in the study area, stable isotopes of δ^18^O and δD were obtained from water samples collected at 27 geothermal wells, water wells, and springs within the study area using hydrogen and oxygen isotope testing methods. The test results are shown in Table 6. After fitting the δD and δ^18^O data from the water sample sites to a linear equation, the calculated R^2^ value of 0.81 indicates a good fit. Comparing the fitted equation with the Chinese meteoric water line and the global meteoric water line (as shown in Fig 30) [38], it can be seen that the three are very similar, and the water sample sites are generally located near the meteoric water lines. thereby confirming that atmospheric precipitation serves as the recharge source for various water bodies within the study area. Sample points AKT1, ZK1, J61, J64, and others are distributed within and around geothermal anomaly areas I, II, and III. This also reflects the common origin of water samples from these three geothermal anomaly areas, all of which receive atmospheric precipitation as a source of replenishment. Furthermore, hydraulic connections exist among them, suggesting that they are controlled by a unified fracture system and constitute an integrated geothermal system.

**Table 6:** Summary of isotope test results.

Number	δ^18^O (‰)	δD (‰)	Remarks
Q9	−11.03	−74.04	Spring
Q13	−11.26	−76.08	Spring
H1	−9.92	−68.29	Surface Water
H2	−10.18	−68.32	Surface Water
J64	−11.89	−81.47	Water Well
Q1	−11.72	−82.08	Spring
J61	−12.08	−84.85	Water Well
J17	−11.79	−82.92	Water Well
H3	−12.81	−89.52	Surface Water
J46	−11.94	−80.31	Water Well
J59	−12.24	−81.87	Water Well
J55	−11.82	−84.92	Water Well
H4	−10.65	−69.97	Surface Water
Q10	−10.59	−71.19	Spring
Q5	−10.91	−76.84	Spring
J57	−10.34	−71.17	Water Well
J62	−10.93	−76.98	Water Well
J56	−11.73	−84.02	Water Well
Q16	−12.06	−82.52	Spring
J21	−11.97	−81.45	Spring
Q4	−11.67	−83.3	Spring
AKT3	−11.59	−80.91	Drilling Hole
ZK3	−11.53	−84.59	Drilling Hole
KC2	−11.59	−83.39	Drilling Hole
AKT1	−13.8	−91.5	Drilling Hole
AKT2	−12.5	−79.5	Drilling Hole
ZK1	−11.78	−85.27	Drilling Hole

At higher elevations, temperatures decrease and the isotopic composition of precipitation becomes less enriched. According to the altitude effect, for every 100 m increase in elevation, the ^18^O content decreases by −0.15‰ to −0.5‰, and the D value decreases by −1‰ to −4‰. Using the altitude effect formula for atmospheric precipitation in China (see Equation 9), the elevations of the geothermal water recharge areas in the study area were calculated and are presented in Table 7.

**Table 7:** Statistical table for calculating geothermal water recharge elevations.

Number	Elevation (m)	δD (‰)	Recharge Elevation (m)
J31	1336.7	−99.4	3620
J35	1318.0	−98.8	3590
AKT3	1315.3	−80.91	2695.5
ZK3	1306.2	−84.59	2879.5
KC2	1344.4	−83.39	2819.5
AKT1	1324.5	−91.5	3225
AKT2	1340.6	−79.5	2625
ZK1	1340.6	−85.27	2913.5


$$\begin{array}{c} \delta D=-\text{0}.\text{02}ALT-\text{27} \end{array}$$ (9)

In the formula: *ALT* represents elevation, in meters.

As shown in Table 7, the recharge elevation ranges from 2625 ~ 3620 m. The Biezhentao Mountain and Alatao Mountain ranges on the northern and southern sides of the study area have elevations ranging from 2400 ~ 4000 m, which is consistent with the estimated recharge elevation range for geothermal water. Given the extremely abundant geothermal water resources in Wenquan County, which require a substantial recharge source, the catchment area of the southern mountainous region is significantly larger than that of the northern region. Furthermore, the northern region faces the sun, receives ample sunlight, and experiences high evaporation rates, resulting in limited recharge from atmospheric precipitation. In contrast, the southern region is shaded from the sun, allowing atmospheric precipitation to infiltrate and recharge the groundwater in large quantities. Therefore, it is concluded that the underground geothermal water originates from the southern Biezhentao Mountain.

## 5. Mechanisms of geothermal field formation

### 5.1. Favorable conduit conditions

Accurate identification of heat-controlling faults is key to understanding the genesis mechanism of geothermal resources. This study employs a variety of methods, including geological data analysis, remote sensing interpretation, geophysical exploration and drilling, and geochemistry, to cross-validate the major heat-controlling faults in the study area.

Analysis of regional geological data reveals the presence of two sets of faults in the study area: one EW-trending and the other NE-SW-trending. These two fault systems intersect in the Shengshan area, forming a “grid-like” network of channels conducive to fluid convergence. The presence of hot spring outcrops at these intersections indicates that both fault systems possess the tectonic conditions necessary to serve as conduits for the ascent of deep thermal fluids. Land surface temperature retrieval results reveal that three temperature anomaly zones are clearly distributed in a linear fashion along the F2, F3-1, and F17 faults. The spatial coupling between the temperature anomalies and the faults indicates that these faults possess good thermal conductivity. CSAMT geophysical profiles show that low-resistivity anomaly zones are present along the N1, N2, N3, N4, N5, and N8 survey lines, respectively. These low-resistivity anomalies result from the fracturing and water-filling of high-resistivity host rock along the faults, and they closely correspond to the surface outcrops of the F2, F2-1, F4, and F5 faults. The presence of these low-resistivity anomalies indicates that the rock masses within these fault zones are fractured, highly porous, and highly permeable, possessing excellent hydraulic and thermal conductivity. Analysis of RQD values from drill cores further validated the locations of the F3-1 fault, F4 fault, and F17 fault group as inferred by geophysical exploration and drilling. The Piper trilinear diagram reveals a high degree of similarity in the chemical composition of geothermal fluids, suggesting they share a common recharge source and evolutionary path, thereby indirectly confirming that the fault system provides a unified migration pathway for geothermal fluids. All five methods consistently indicate that F2, F3-1, and F17 are the primary heat-controlling faults in the study area. Among these, F2 is a regional heat-controlling fault, while F3-1 and F17 are water- and heat-conducting faults; their combination constitutes the main migration pathways for geothermal fluids.

### 5.2. Favorable reservoir conditions

Geothermal measurement data show that at depths of 400 ~ 430 m in Well KC2, the temperature suddenly increased from 60.5°C to 69.5°C, indicating that this depth interval is the center of a heat-conducting structure. CSAMT geophysical profiles reveal that the low-resistivity anomaly zone is primarily distributed between depths of 420 ~ 1855 m, which is a favorable location for a thermal reservoir. The silica temperature scale estimates an average thermal reservoir temperature of 132.4°C; based on the regional geothermal gradient, this corresponds to a depth of approximately 1200 ~ 1800 m. The K-Mg temperature scale estimates values ranging from 71.4 ~ 102.0°C in some samples from the equilibrium zone, which is consistent with the observed trends in bottom-hole temperature. Based on a comprehensive assessment, the geothermal reservoir in the study area is located at depths of 800 ~ 1500 m, primarily within fault and fracture zones, with a reservoir temperature of approximately 120 ~ 140°C, classifying it as a medium-to-low-temperature zonal geothermal reservoir. The vertical isotherms of the geothermal field form two upward bulges in the western and central sections of Mount Sheng, corresponding to the location of the F17 fault, indicating that geothermal fluids rise along the fault from the basement and exhibit a zonal distribution.

### 5.3. Stable fluid conditions

**Fig 31:**
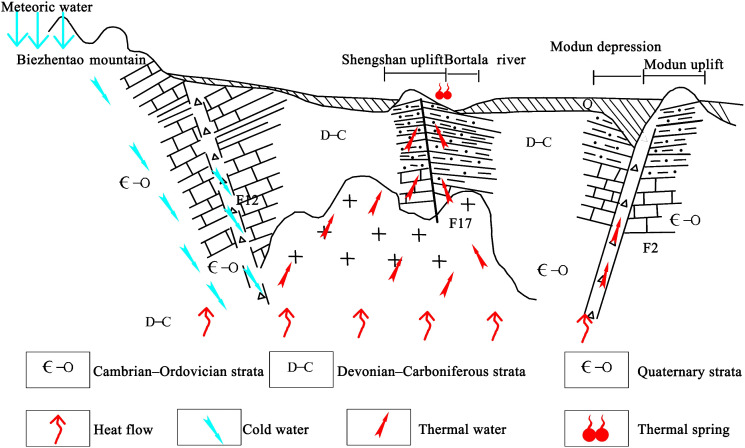
Schematic diagram of groundwater circulation pathways in the study area. The blue and red arrows in the figure represent the circulation paths of cold water and hot water, respectively, within this system.

Results of hydrogen and oxygen isotope analysis show that all water sampling sites fall near the global meteoric water line, indicating that the geothermal water, spring water, and surface water in the study area all originate from meteoric water. Calculations of recharge elevation indicate that the geothermal water recharge elevation ranges from 2625 ~ 3620 m. Considering that the southern mountainous region has a larger catchment area and lower evaporation rates, it is inferred that the southern Biezhentao Mountain is the primary recharge area for the geothermal water. The Piper trilinear diagram shows a high degree of overlap among the eight groups of water samples, all of which belong to the SO_4_-Na hydrochemical type, indicating that the geothermal waters share the same recharge source and hydrogeochemical evolution pathway. The Schoeller diagram shows elevated SO_4_^2-^ concentrations and low Ca^2+^ concentrations, indicating that the aquifer has been subjected to hydrothermal alteration. Concurrently, CO_2_ escapes as the groundwater rises, triggering decarbonation and the formation of CaCO_3_ precipitates. This process further confirms that the geothermal water has undergone deep circulation and hydrothermal modification. Based on a comprehensive analysis of topography, fault structures, and hydrochemical characteristics, the groundwater circulation pathway in the study area can be summarized as follows: Atmospheric precipitation infiltrates in the southern Biezhentao Mountain and migrates deepward along fault fracture zones; at depth, it undergoes heat exchange with mantle heat flow to generate geothermal water; the geothermal water ascends along the F2, F3-1, and F17 fractures, eventually emerging as hot springs near the fracture zone. The groundwater circulation pathway in the study area is shown in Fig 31.

### 5.4. Stable heat source conditions

Calculations indicate that the terrestrial heat flow value in the study area is 320 mW/m^2^, which is significantly higher than the average terrestrial heat flow value at the northwestern margin of the Junggar Basin in Xinjiang, suggesting that the study area has an abnormally high terrestrial heat flow background. Calculations of the radiogenic heat production rate from rock radioactivity show that the overall average rock heat production rate for the three major rock types in the study area is 1.99 μW/m^3^, contributing 1.99 mW/m^2^ to the terrestrial heat flow per kilometer of thickness. This indicates that radiogenic heat production is one of the stable heat sources for the geothermal field, but not the primary one; the heat flux from the deep mantle plays a dominant role.

### 5.5. Comprehensive genesis analysis of the geothermal field

**Fig 32:**
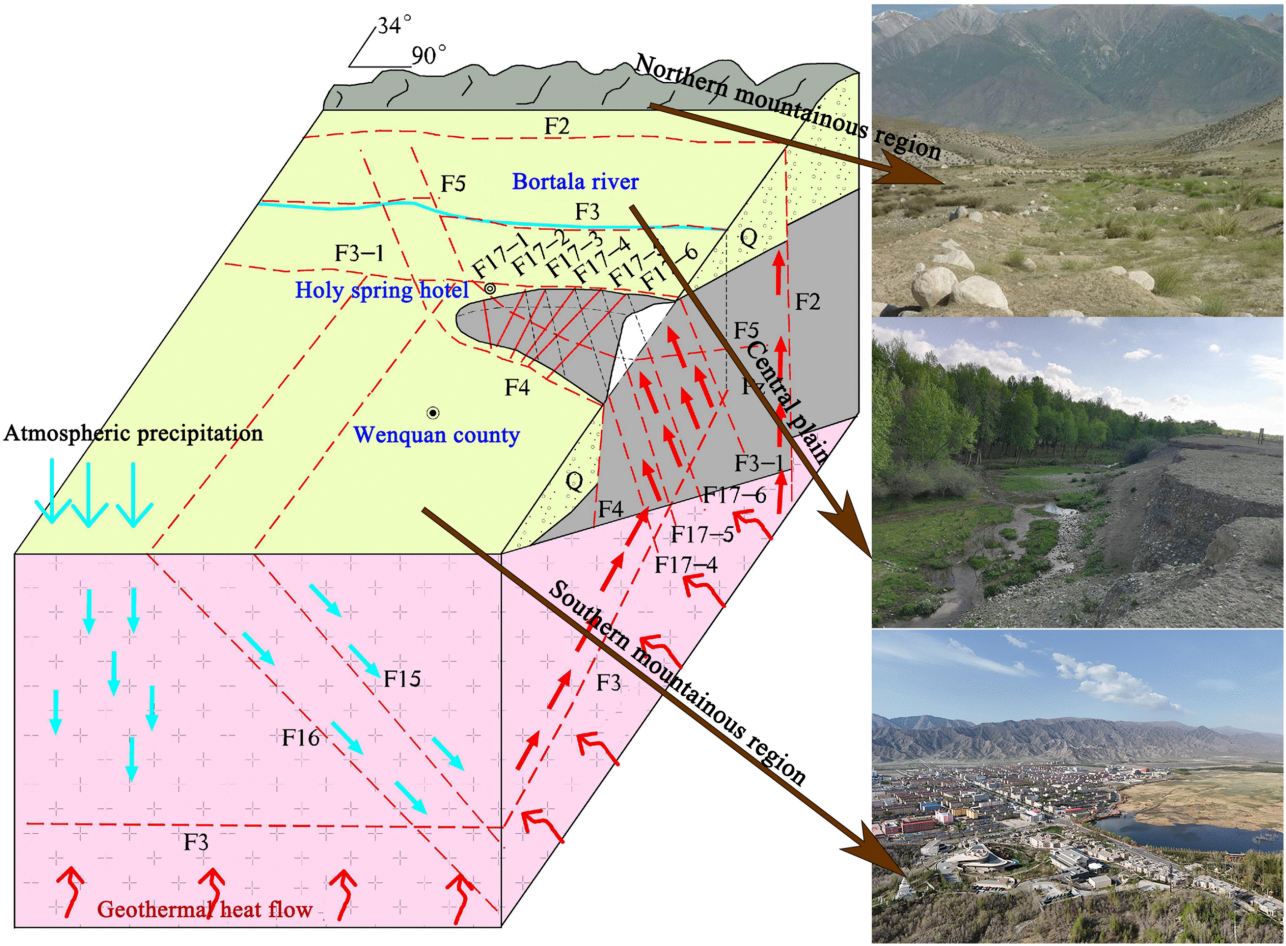
Geothermal genesis model of the study area. The image on the right shows a photo taken on-site; the blue and red arrows indicate the circulation paths of cold water and hot water, respectively, within the geothermal system.

Based on a comprehensive analysis of the above research findings, a conceptual model of the genesis mechanism of geothermal resources in the study area was developed (as shown in Fig 32). The geothermal field is situated in the intermontane valley between the northern and southern mountains. The overall terrain slopes from west to east. Under the control of major structures, including the heat-controlling F2 fault and the water- and heat-conducting faults F3-1 and F17, the geothermal system is recharged by atmospheric precipitation infiltrating from the southern Biezhentao Mountain area, forming cold groundwater. This water converges along the fault fracture zones, infiltrates downward to undergo deep circulation, and exchanges heat convectively with deep heat sources (mantle-derived heat flow), thereby forming geothermal water. Finally, it rises along the F2, F3-1, and F17 faults and emerges at the surface in the form of hot springs.

## 6. Conclusions and perspectives

(1) Geothermal anomalies in the study area are significantly controlled by fault structures, with an average geothermal gradient of 11.53°C/100 m and a terrestrial heat flow value of 320 mW/m2, indicating favorable heat flow background conditions. Radiogenic heat production (average 1.99 μw/m3) provides a secondary heat source for the geothermal field, while deep mantle heat flow constitutes the primary heat source.
(2) Faults F2, F3-1, and F17 are the main heat-controlling faults in the study area, among which F2 is a regional heat-controlling fault, while F3-1 and F17 are water-and heat-conducting faults. The fracture zones formed by their intersections exhibit high porosity and strong permeability, constituting the main migration pathways for geothermal fluids and the primary reservoir space. The geothermal reservoir is classified as a medium-low temperature belt-type reservoir, with an estimated temperature of approximately 120 ~ 140°Cand burial depth of 800 ~ 1500 m, indicating considerable development potential.
(3) The geothermal water is of the SO4-Na type and is replenished by atmospheric precipitation infiltrating the mountainous areas of the Biezhen Taoshan Range in the south. The geothermal water undergoes heating through deep circulation and intense water-rock interactions, then rises along heat-conducting faults to emerge as hot springs.
(4) The mineralization pattern of the Hot Spring County geothermal field is as follows: controlled by the intersection zone of NE-and NW-trending faults, it extends in a long, narrow strip along the fault zone; the geothermal field exhibits a three-layer vertical structure of “temperature-varying layer—constant-temperature layer—temperature-increasing layer,” with the geothermal gradient decreasing from the center of the fault zone toward both sides.
(5) This study has the following limitations: the distribution of geothermal wells and sampling points is uneven, with most concentrated in the Shengshan uplift area and fewer in the western mountainous areas and eastern plains; the hydrochemical and isotopic data are based on single sampling events and lack seasonal dynamic monitoring; and no numerical simulations were conducted, so the deep circulation pathways and water-rock interaction mechanisms remain conceptual inferences. Future work will focus on adding temperature measurement points, establishing a dynamic monitoring system for geothermal fluids, and conducting numerical simulations to further validate the conceptual model.

## Supporting information

S1 Data Raw Data Table of RQD Values for Well AKT1. (TIF)

S2 Data Raw Data Table of RQD Values for Well AKT2. (TIF)

S3 Data Raw Data Table of RQD Values for Well AKT3. (TIF)
